# Measurement of sludge-water interface based on adjustable peak point of ultrasonic echo energy

**DOI:** 10.1371/journal.pone.0332781

**Published:** 2026-07-23

**Authors:** Miao Li, Yang Miao, Fengying Zhou, Yongqiang Yang, Huan Huang

**Affiliations:** 1 School of Mechatronic Engineering, Beijing Polytechnic College, Beijing, China; 2 College of Mechanical and Energy Engineering, Beijing University of Technology, Beijing, China; Monash University, AUSTRALIA

## Abstract

To address the escalating water resources consumption, the need for intelligent automation in urban sewage treatment has become imperative. However, existing ultrasonic sludge interface instruments have been found to exhibit poor environmental noise suppression and low identification accuracy of mud-water interface, thereby necessitating a new approach. This paper analyzes the problems and proposes a mud-water interface monitoring method based on extraction of ultrasonic energy peak point. The method involves an array ultrasonic system structure comprising multiple ultrasonic transmitting and receiving elements, median value average filtering to suppress peak noise interference, and interval maximum method to separate the target peak point from residual noise. The proposed method was tested using a high-precision ultrasonic probe in Beijing Ma-Fang Sewage Treatment Plant and corrected using a fault diagnosis method with 3σ threshold. Results demonstrated a high measurement accuracy with a mean deviation of less than 2% and a precision variance below ±0.02 m. Compared to conventional ultrasonic and optical methods, this approach exhibited superior robustness in turbid environments, fully meeting the technical requirements of sludge thickness monitoring.

## 1. Introduction

During primary wastewater treatment, raw wastewater undergoes initial filtration and sedimentation to remove larger suspended solids and particulate matter [1]. Subsequently, the wastewater is conveyed via lift pumps to primary settling tanks, where an extended sedimentation process facilitates the separation and concentration of settleable organic solids. Sludge blanket level—a critical parameter governing sedimentation efficiency—is monitored in real-time using sludge interface meters, a technology enabled by advancements in online sensing [2,3]. Since the 1970s, pulse-infrared detection systems have been implemented for interface monitoring in sedimentation and thickening tanks by environmental protection firms in developed nations [4]. However, due to limitations in online monitoring technology, domestic wastewater treatment plants in many regions still predominantly rely on manual methods such as rod insertion and visual inspection for sludge thickness assessment [5]. These manual techniques are subject to inaccuracies, operational safety risks, and low efficiency.

In recent years, considerable progress has been achieved in the development of ultrasonic, optical, and hybrid sensing technologies for sludge-interface detection and wastewater monitoring. Mukherjee et al. introduced a self-calibrating ultrasonic system for liquid–liquid interface detection, demonstrating improved signal stability but limited applicability in turbid media [6]. Zhang et al. proposed an echo-energy balance technique for clear liquid level measurement, which, although precise, exhibited reduced accuracy in multiphase suspensions [7]. More recently, Yao et al. and Xu et al. incorporated temperature-compensated ultrasonic algorithms and numerical simulations to analyze acoustic propagation in complex two-phase flows, providing valuable insights into echo attenuation mechanisms [8–9]. Optical and infrared techniques have also been explored for interface monitoring [10], yet their performance deteriorates in opaque sludge environments due to high turbidity and scattering. Beyond detection hardware, intelligent filtering and adaptive threshold algorithms have been applied to environmental signal processing, suggesting that integrated data-driven correction frameworks may enhance measurement accuracy [11]. Despite these advances, existing approaches largely target well-defined liquid–air or oil–water boundaries and seldom address the dynamic, diffuse sludge–water interface characteristic of municipal sedimentation tanks. The present study aims to fill this gap by introducing an ultrasonic detection methodology based on the adjustable peak point of echo energy, incorporating adaptive filtering and fault-threshold correction to achieve reliable, real-time sludge thickness estimation under variable operational conditions.

In the domain of ultrasonic liquid interface detection, extant research has predominantly emphasized interfaces exhibiting pronounced layering phenomena, such as liquid-air or water-oil boundaries, where significant disparities in acoustic impedance and reflectivity facilitate straightforward discrimination from ambient noise [11,12]. Conversely, within sewage settling tanks, the interface between the clear water layer and the suspended layer manifests as a heterogeneous turbid liquid surface devoid of distinct stratification. Such complex multiphase dynamic behaviors and the requirement for advanced modeling are similarly critical in broader environmental engineering contexts, such as municipal solid waste incineration processes [13,14]. This complexity arises from ubiquitous interfering substances (e.g., sludge, bubbles, and particulates) and environmental variables like temperature fluctuations, which markedly degrade detection stability, accuracy, and reliability relative to conventional liquid level measurements [15,16]. Rana et al deployed an ultrasonic transducer directly into the mud-water interface, coupling it with a winch mechanism to enable vertical tracking via feedback-controlled motion, a methodology analogous to cable-driven probe systems detailed in [17]. Yao et al engineered an ultrasonic detection system leveraging microcontroller programming and temperature compensation algorithms to mitigate measurement errors [18]. Similarly, Singhal et al conducted direct ultrasonic assessments of the target interface by implementing a shielded measurement cylinder, an approach resonating with controlled-environment techniques [19].

To enhance the accuracy and efficiency of online monitoring for sewage sedimentation tanks, this study introduces a novel methodology for sludge thickness measurement utilizing ultrasonic transit-time principles. The approach transforms ultrasonic signals into energy peaks, extracts distinctive characteristics of the sludge-water interface, calculates sludge thickness, and integrates a data compensation and correction mechanism optimized for sedimentation tank conditions. This methodology establishes a theoretical foundation for advancing intelligent urban sewage treatment systems.

## 2. Principle of Detection

### 2.1. echo signal

**Fig 1:**
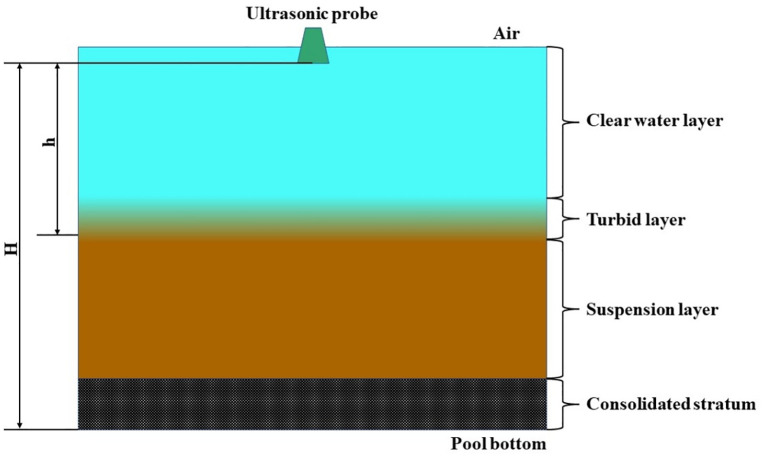
Ultrasonic probe installation and water layer.

Ultrasonic waves are mechanical sound waves with frequencies exceeding 20 kHz, characterized by superior directionality, strong penetration capabilities, and high measurement accuracy. Common methodologies for ultrasonic measurement include the echo envelope method [20], least squares optimization [21], and the transit-time method (also known as time-of-flight, ToF), the latter being widely adopted for its practicality. Furthermore, as ultrasonic waves propagate through diverse media, measurement systems are categorized into air-coupled, liquid-coupled, and solid-coupled configurations [22,23]. Owing to the principle that higher frequencies yield finer ranging resolution, and considering the markedly lower attenuation of ultrasonic waves in liquids compared to gases, this study employs a liquid-coupled operational mode. The ultrasonic transducer is submerged and oriented perpendicular to the pool bottom to facilitate measurement, leveraging the near-total reflection occurring at the liquid-air interface. The sensor installation configuration is detailed in Fig 1.

The fundamental principle of ultrasonic measurement operates as follows: Ultrasonic pulses emitted by the transducer propagate through a medium and undergo continuous reflection at material interfaces. A receiver captures these reflected signals at predetermined time intervals, and an electronic processor converts the time-of-flight (TOF) data into the distance between the transducer and the target surface using the relationship, where $d=\frac{\text{1}}{\text{2}}ct$ is the speed of sound and t is the round-trip time. This pulse-echo methodology, commonly termed the time-of-flight principle, employs the reflection formula detailed in reference [22].


$$\begin{array}{c} D\left(z_{\text{1}},z_{\text{2}}\right)=\frac{4z_{\text{2}}z_{\text{1}}}{(z_{\text{2}}-z_{\text{1}}{)}^{\text{2}}} \end{array}$$ (1)

where R denotes the reflection coefficient, defined as the ratio of the intensity of the reflected wave to that of the incident wave; D represents the transmission coefficient, expressed as the ratio of the intensity of the transmitted wave to that of the incident wave; and Z signifies the acoustic impedance of the medium, characterized as the product of the medium density and the wave velocity [24].


$$\begin{array}{c} z_{i}=\rho_{i}C_{i} \end{array}$$ (2)

The reflection coefficient is governed by the difference in acoustic impedance between adjacent media. A large impedance mismatch, such as at the tank bottom (e.g., concrete,Zbottom≫Zwater), produces a strong, sharp echo (the first peak). Unlike the abrupt impedance discontinuity at the tank bottom, the sludge-water interface is a heterogeneous transition zone. The acoustic impedance (Z = \rho c) in this zone changes gradually due to the varying volumetric concentration of suspended sludge flocs. As the ultrasonic wave penetrates this transition layer, the change in local density (\rho) induces continuous scattering and absorption, which dissipates the acoustic energy. This scattering mechanism explains why the echo from the sludge interface (the secondary peak) manifests as a broader, lower-amplitude signal compared to the specular reflection from the concrete bottom. The proposed energy-based method is explicitly designed to identify this dispersed energy signature.

In accordance with the physical principles governing acoustic wave propagation in liquid media, variations in temperature and pressure exert a direct influence on the speed of sound. This relationship arises from the interdependence of fluid density, compressibility, and elastic properties with thermodynamic conditions. For aqueous systems, the following empirical formulation [25] characterizes this relationship:


$$\begin{array}{c} C\left(P,T\right)=1402.7+488T-482T^{\text{2}}+135T^{\text{3}}+(15.9+2.8T+2.4T^{\text{2}})P/100 \end{array}$$ (3)

where P is the standard pressure in bar, andT is the temperature parameter.

Let t be the time of flight for the ultrasonic wave to propagate from the sensor to the target interface and return to the sensor. The distance L from the sensor to the target is then calculated as:


$$\begin{array}{c} L\left(C,t\right)=\frac{C\left(P,T\right)t}{\text{2}} \end{array}$$ (4)

**Fig 2:**
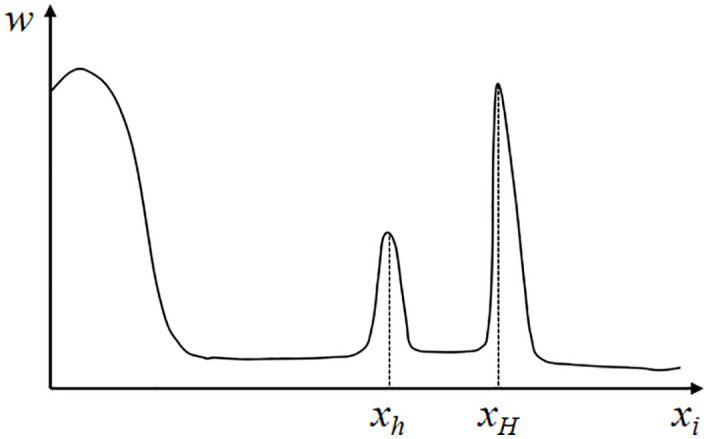
The ideal line shape of the echo signal.


The receiver collects echo signals at fixed timeτ intervals, so the continuous liquid environment is divided into multiple overlapping micro-layers. For any layer, the distance L to the sensor is described by the discrete position point $x_{i}$ on the horizontal axis in Fig 2:

$$\begin{array}{c} L_{i}=C\times \tau \times x_{i} \end{array}$$ (5)

where $x_{i}$ represents the i-th position point, and $L_{i}$ denotes the distance from the layer corresponding to the i-th position point to the sensor. The entire liquid environment is described as:


$$\begin{array}{c} \left\{\left(w_{i},x_{i}\right)\right|w_{i}=f\left(x_{i}\right)\} \end{array}$$ (6)

where $w_{i}$ represents the ultrasonic intensity at the i-th position point. The intensity $w_{i}$ and position point$x_{i}$ can form a discontinuous curve. The system will upload this data in the form of a one-dimensional array for subsequent calculations. The ideal linear shape of the echo signal curve is shown in Fig 2. Theoretically, prominent reflected energy will be generated at the pool bottom $x_{H}$ and the mud-water interface $x_{h}$ positions.

The initial segment of the curve displays persistent high-energy oscillations, defining the system’s blind zone. This phenomenon is primarily caused by two factors: (1) the prolonged resonant vibration (ringing) of the transducer’s piezoelectric crystal after the excitation pulse, and (2) the saturation of the receiver circuitry by the strong transmitted signal. Together, these effects create a period during which weak returning echoes are completely obscured. The length of this blind zone was empirically determined to be approximately 200 sampling points (~0.8 m). To ensure this blind zone does not impact the accuracy of sludge thickness measurement, the sensor was installed at a fixed depth such that the entire region of interest (from 0.5 m to 3 m) lies entirely outside this zone. Furthermore, the data processing algorithm automatically discards all data within the first 230 points, providing a conservative buffer against any blind zone interference.

### 2.2. Configuration of Measurement System

**Fig 3:**
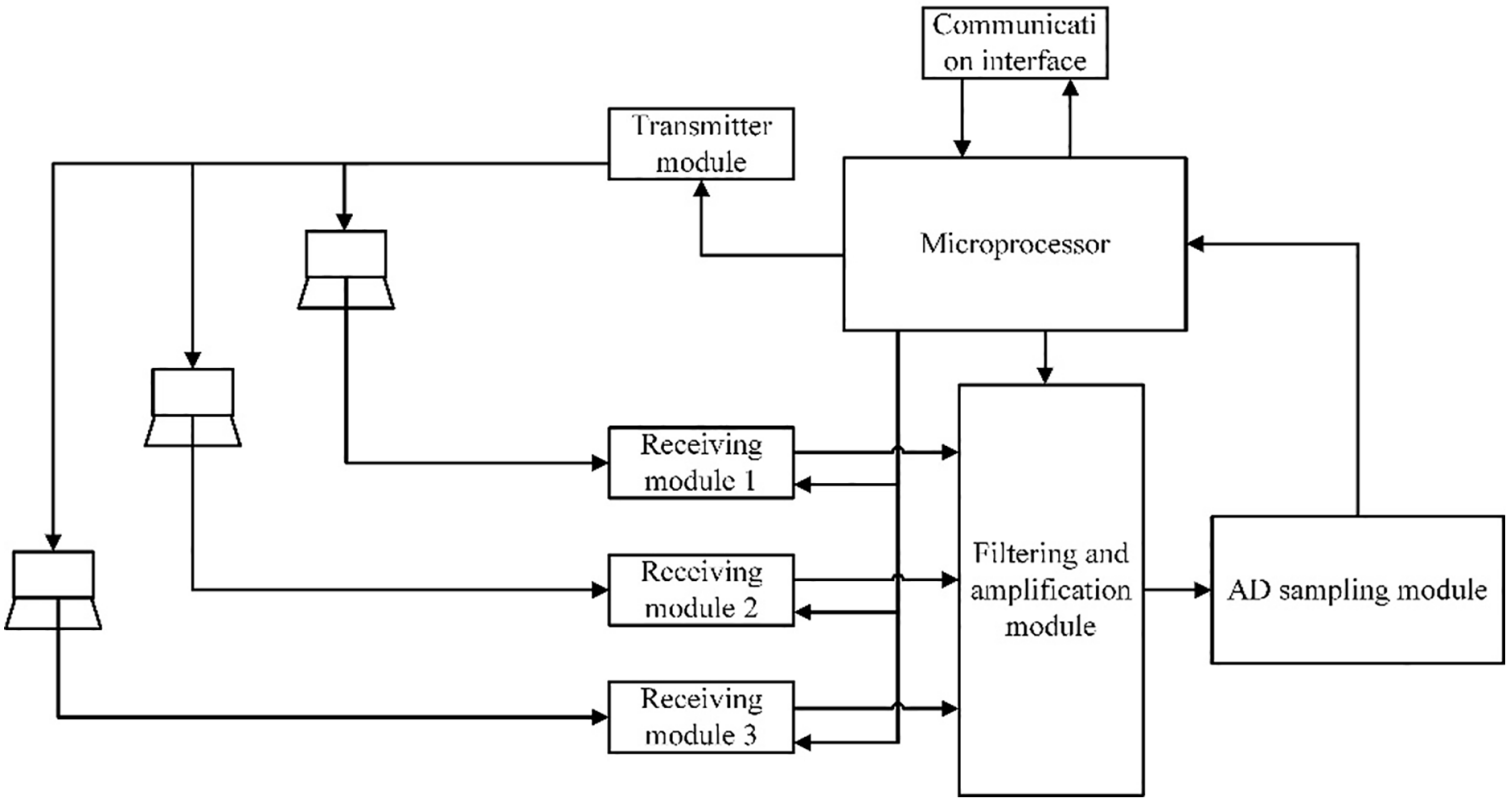
Block diagram of sludge interface instrument system.

The ultrasonic transducer array consists of three independent 1 MHz piezoelectric transducers arranged in a center-symmetric triangular pattern with a spacing of 10 cm between adjacent elements. This configuration ensures spatial diversity and redundancy. (see Fig 3). This configuration aligns with standard ultrasonic sensor designs where piezoelectric elements serve dual roles. Signal driver circuits: Separate transmitter and receiver driver modules generate excitation pulses for the transmitters and condition received signals. This design reflects dedicated driver circuitry requirements for piezoelectric transducers.Filtering stage: Output from the receiver module undergoes signal conditioning through a dedicated filter module to enhance signal integrity.Data acquisition: An analog-to-digital conversion (ADC) module samples the filtered analog signal for digital processing. Control unit: A microprocessor coordinates system operation by managing driver circuits, ADC module, and communication interfaces. The microprocessor (ARM Cortex-M4) utilizes hardware timers and DMA to ensure precise timing and synchronization across all three transducer channels. A shared master clock minimizes jitter. To reduce noise and interference, shielded cables are used for all signal connections, and the receiver incorporates a programmable gain amplifier (PGA) alongside an anti-aliasing filter. Digital median averaging further suppresses transient noise in software.This central control architecture corresponds to microprocessor-based ultrasonic system designs. Fig 3 illustrates the overall system block diagram, demonstrating the integration of these functional modules.

The choice of a multi-element transducer configuration, as opposed to a phased array, was made based on a balance of performance, cost, and complexity for this specific application. While a phased array could potentially offer superior spatial resolution through electronic beam focusing and steering, providing a finer-grained acoustic image of the interface topology, the multi-element approach provides substantial practical advantages. The spatial diversity afforded by independent probes effectively captures the non-planar nature of the sludge blanket and mitigates the impact of local anomalies, which is crucial in the turbulent environment of a sedimentation tank. Given the successful accuracy demonstrated by the signal processing algorithms developed in this work, the multi-element system presents a more cost-effective and immediately deployable solution. The increased electronic and computational complexity of a phased array, while a promising avenue for future research, was not deemed necessary for achieving the core objective of reliable interface depth measurement in this study.

### 2.3. Influence of Temperature and Pressure on Sound Speed and System Compensation

The propagation speed of sound is a critical parameter governing the accuracy of ultrasonic distance measurement. In liquid media, sound speed is subject to significant variation induced by changes in temperature and pressure, as characterized by the empirical Wilson’s equation:


$$\begin{array}{c} \left(T,P)=c_{\text{0}}+a(T-T_{\text{0}})+b(P-P_{\text{0}}\right) \end{array}$$

where $c_{\text{0}}$ denotes the sound speed at reference temperature $T_{\text{0}}$ and pressure $P_{\text{0}}$, while a and b represent the temperature and pressure coefficients of sound speed, respectively. In aqueous systems, sound velocity typically increases within a range of 3–5 m/s per 1°C rise in temperature, and approximately 0.016 m/s per 1 bar increase in pressure.

Within the sludge-water medium, the presence of suspended solids introduces further complexity to sound propagation. Variabilities in particle concentration, size distribution, and bulk density collectively influence the effective sound speed. Uncompensated deviations in sound velocity can therefore propagate into substantial errors in the computed sludge thickness.

It should be noted that while direct, in-situ measurement of sound velocity—for instance, via a fixed-path reflector or multi-frequency phase analysis—represents a potentially more accurate approach for heterogeneous media, the present study adopts the empirical Wilson equation as a pragmatic and well-validated engineering solution. In the relatively stable, low-turbidity supernatant of a secondary sedimentation tank, temperature is the dominant variable affecting sound speed, with salinity and hydrostatic pressure variations being secondary. Under these conditions, the temperature-compensated Wilson model provides an accuracy of ±2–3%, which is within the operational tolerance for sludge blanket monitoring. Furthermore, the subsequent implementation of the 3σ adaptive fault-threshold correction (Section 3.4) is designed to dynamically compensate for residual systematic and random errors, including those arising from mild, unaccounted-for sound speed fluctuations. The integration of a direct in-situ velocimeter is identified as a strategic enhancement for future system refinement.

To mitigate these effects, the measurement system incorporates the following compensation mechanisms:

1. Real-Time Temperature Sensing: A temperature sensor is collocated with the ultrasonic probe assembly to provide direct measurement of the medium temperature T.
2. Pressure Consideration: Given the open-atmosphere configuration of the sedimentation tank, hydrostatic pressure variations are negligible relative to temperature effects; compensation is thus primarily focused on thermal drift.
3. Adaptive Sound Speed Calibration: The instantaneous sound speed c(T) is dynamically computed using Wilson’s equation, based on the acquired temperature T. This value is subsequently utilized in the time-to-distance conversion per Equation (4):

$$\begin{array}{c} d=\frac{c(T)\cdot t}{\text{2}} \end{array}$$

4. Multi-Channel Data Fusion: The integration of measurements from three spatially distributed probes provides inherent averaging, reducing the impact of localized thermal gradients on the final sludge thickness estimation.The present system incorporates temperature compensation by placing a temperature sensor near the ultrasonic probe assembly. The measured local temperature is used as the input to the Wilson equation, allowing the sound speed to be dynamically updated during the time-of-flight conversion process. This correction reduces errors caused by bulk temperature variation during field operation. However, this compensation mainly accounts for the local measured temperature and cannot fully resolve continuous temperature gradients along the entire acoustic propagation path. This limitation motivates the future integration of direct in situ sound-speed calibration.

During field experiments, the recorded water temperature ranged between 12–14°C under an ambient pressure of ~1 bar, yielding a calculated sound speed interval of 1454.7–1462.1 m/s. The implementation of the described compensation strategy enabled the system to maintain consistent measurement accuracy across this temperature range, with observed deviations remaining below 2% relative to manual reference measurements.

Potential errors associated with the Wilson equation mainly originate from the assumption that the medium can be represented by a single temperature- and pressure-dependent sound speed. In real sludge-water environments, temperature gradients, salinity changes, suspended solids concentration, bubbles, and local density variations may cause the actual sound speed to deviate from the value calculated by the empirical equation. Temperature gradients are particularly important because sound velocity in aqueous media generally changes by approximately 3–5 m s ^−^ ^1^ for each 1 °C variation. As the measured distance is proportional to sound speed, these deviations may lead to propagated errors in sludge-depth estimation, especially over meter-scale acoustic paths.

### 2.4. Analysis of environmental factors and system mitigation strategies

In practical wastewater treatment environments, the propagation of ultrasonic signals is susceptible to interference from various environmental factors, primarily including biofouling on the transducer surface, suspended solids concentration, salinity variations, temperature gradients, and bubbles. Specifically, the accumulation of biofilms and contaminants can alter the acoustic impedance at the sensor interface and increase signal attenuation. These factors collectively cause changes in sound velocity, signal attenuation, or scattering, thereby affecting the accuracy of sludge-water interface detection. This study addresses these challenges through theoretical analysis and specific system design features.

(1) Impact of Suspended Solids and Mitigation:High concentrations of suspended solids enhance ultrasonic scattering and energy attenuation, leading to a reduced signal-to-noise ratio (SNR) in the echo signal and obscuring the interface peak. The attenuation coefficient $\alpha_{total}$ for ultrasound in a medium containing suspended particles can be approximated as the sum of absorption and scattering attenuation:


$$\begin{array}{c} \alpha_{total}=\alpha_{a}+\alpha_{s}\approx k_{\text{1}}f+k_{\text{2}}C_{ss}f^{\text{4}} \end{array}$$

where $\alpha_{a}$ and $\alpha_{s}$ are the absorption and scattering attenuation coefficients, respectively, f is the ultrasonic frequency, $C_{ss}$ is the suspended solids concentration, and $k_{\text{1}}$, $k_{\text{2}}$ are coefficients related to the medium’s properties. This equation shows that attenuation increases significantly with both suspended solids concentration and frequency. To suppress this interference, the system employs a moderate-frequency probe in its hardware design, balancing resolution against excessive attenuation. Furthermore, in signal processing,the median average filtering (detailed in Section 3.2, Equation 7) and the interval maximum method (Section 3.3, Equations 8–10) and waveform morphology analysis are employed to effectively remove transient pulse noise and adapt to variations in ultrasonic reflectivity caused by changing sludge density and particle size distribution. The multi-element ultrasonic array enhances the identification of the true interface signal through spatial diversity.

(2) Impact of Salinity and Temperature, and Compensation:Variations in salinity S and temperature T in the wastewater alter the medium density and acoustic impedance, thus affecting the sound velocity c, which ultimately introduces ranging errors $\Delta d=\frac{\text{1}}{\text{2}}c\cdot \Delta t$. Sound velocity c is a function of T, S, and static pressure P. Building upon the Wilson empirical formula (Equation 3), a salinity correction term can be introduced:


$$\begin{array}{c} \begin{array}{c} c\left(T,S\right)=c_{\text{0}}+\Delta_{CT}+\Delta_{CS}\mathrm{\backslash hfill}\\ \;\;\;\approx 1449.2+4.6T-0.055T^{\text{2}}+0.00029T^{\text{3}}+(1.34-0.010T(S-35)\mathrm{\backslash hfill} \end{array} \end{array}$$

where $c_{\text{0}}$ is a reference sound velocity. The system incorporates real-time temperature compensation (based on Equation 3) via a built-in temperature sensor. Although salinity is not directly measured, its effect on sound velocity in a stable wastewater treatment environment can be considered a slow-varying parameter. The error introduced by salinity is partially accounted for within the system’s error margin and can be subsequently identified and corrected by the proposed fault threshold correction method (detailed in Section 3.4, see Equations 16–18).

(3) Suppression of Bubble and Flow Disturbances:Bubbles and flow disturbances in the sedimentation tank generate random noise peaks, the amplitude of which can potentially mask the true interface echo. The system confines the peak search range using the interval maximum method (Section 3.3) to avoid misinterpreting bubble reflections as interface signals. Concurrently, the 3σ threshold-based fault diagnosis (Section 3.4, Equation 16) is applied to assess the plausibility of consecutive measurement results. This method’s effectiveness relies on the assumption that anomalies caused by transient disturbances like bubbles are low-probability events, whose statistical characteristics follow a Gaussian distribution. For any measurement sequence $\left\{d_{\text{1}},d_{\text{2}},...,d_{n}\right\}$, its mean $\mu_{d}$ and standard deviation $\sigma_{d}$ define the reasonable fluctuation range $\left[\mu_{d}-\text{3}\sigma_{d},\mu_{d}+\text{3}\sigma_{d}\right]$. Data points falling outside this range are identified as outliers induced by strong interference and are corrected, thereby ensuring the robustness of the output.To further clarify the signal processing chain and its robustness against noise: the raw ultrasonic echo signal first passes through an analog front‑end consisting of a programmable gain amplifier (PGA) and an anti‑aliasing filter (cutoff frequency 1.2 MHz). The digitized signal (1 MHz sampling, 12‑bit resolution) then undergoes median‑average filtering (window size = 50 points) to suppress impulsive noise caused by suspended solids and microbubbles. After filtering, the global maximum method identifies the tank bottom peak, and the interval maximum method (search window = 250 points near the expected interface location) extracts the sludge‑water interface peak. Finally, the 3σ adaptive fault threshold algorithm corrects outlier readings. Under typical operating conditions (SNR = 8–15 dB for the interface echo), this chain limits the peak positioning error to <±2 cm, as validated by field comparisons against manual rod measurements.

In summary, the system addresses the impact of suspended solids, salinity, and bubble disturbances on measurement accuracy through theoretical modeling of the influencing mechanisms and a co-design of hardware selection and signal processing algorithms (filtering, peak extraction, threshold diagnosis), thereby enhancing adaptability and reliability in real-world wastewater treatment scenarios.

## 3. Methodology

To accurately extract the highest peak (H) and the second highest peak (h) from ultrasonic echo signals, this paper adopts a strategy combining the global maximum method and the interval maximum method. The global maximum method is used to identify the point of highest global energy, corresponding to the water surface interface with the strongest reflection. The interval maximum method, on the other hand, searches for local maxima within a preset depth range to determine the sludge-water interface position, thereby avoiding the misidentification of non-target reflections (such as surface scum or floating solids) as the second highest peak. The length of this search interval is determined based on system calibration experiments: by collecting a large number of echo signals under known sludge thickness conditions, the time delay distribution range of the sludge interface echoes is statistically analyzed. Combined with the sensor installation position and pool structure, a reasonable window is set, with an additional ±3 cm safety margin to accommodate fluctuations in actual operating conditions. This dynamic search mechanism ensures that the target peaks lie within a depth range that conforms to physical significance, effectively excluding interference sources such as pool bottom reflections or structural clutter.

To address potential peak overlap or closely spaced echoes (e.g., when the suspended solids layer and sludge layer are close), this paper introduces waveform morphology analysis and time-series continuity constraints for auxiliary discrimination. By analyzing features such as the rising edge slope, echo width, and symmetry of candidate peaks, interface reflection signals are distinguished from scattering noise. Simultaneously, leveraging the slow variation characteristic of sludge thickness, consistency checks are performed on detection results from adjacent time points to prevent abrupt misjudgments. When potential peak overlap is detected, sub-sampling techniques such as parabolic interpolation are employed to enhance the resolution of time delay estimation, thereby more accurately distinguishing closely adjacent echoes. This comprehensive strategy significantly improves the robustness and accuracy of peak identification while ensuring computational efficiency, guaranteeing that the extracted peaks reliably correspond to the actual physical interfaces.

However, the interval maximum strategy also has inherent limitations. Because the search interval is determined by calibration results and tank geometry, it should not be regarded as a universally fixed parameter; it needs to be updated when the sensor installation depth, tank structure, or dominant sludge-layer distribution changes. If a strong non-target echo appears inside the calibrated interval and exhibits waveform morphology similar to the real sludge-water interface echo, the method may still misclassify the candidate peak, particularly under very low SNR or severe aeration/turbulence conditions. Therefore, the interval maximum method is used in this study as a physically constrained primary extractor rather than an independent amplitude-only decision rule, and its output is jointly verified by waveform morphology, adjacent-time continuity, and the subsequent 3σ fault-threshold correction.

For precise calculation of sludge thickness, the highest peak $x_{H}$ and second highest peak $x_{i}$ must be accurately identified from Fig 2. However, sludge sedimentation tanks operate under intricate hydrodynamic conditions with significant disturbances, necessitating robust data processing methods to discern meaningful signals from background noise.

### 3.1. Signal Processing Chain Overview

The complete signal processing procedure consists of four sequential stages:

1. Analog conditioning: The received echo signal is amplified by a programmable gain amplifier (PGA) with gain adjustable from 20 dB to 60 dB, then low‑pass filtered (anti‑aliasing, cutoff 1.2 MHz) before analog‑to‑digital conversion (12‑bit ADC, 1 MHz sampling rate).
2. Digital filtering: Median‑average filtering (detailed in Section 3.2) removes impulsive noise while preserving the sharpness of the sludge‑water interface echo. A window size of 50 sampling points is empirically determined as optimal.
3. Peak extraction: The global maximum of the filtered signal locates the tank bottom (first peak). The interval maximum method (Section 3.3) searches within a predefined depth window (250 points wide, positioned just before the expected sludge blanket depth) to identify the sludge‑water interface as the second highest peak.
4. Post‑processing correction: The 3σ adaptive fault threshold method (Section 3.4) dynamically validates and corrects output depth values against a fused Gaussian model, eliminating transient anomalies.

The effect of signal noise varies across stages: impulsive noise is mainly rejected by the median filter; Gaussian electronic noise is partially suppressed by averaging; and persistent broadband interference is mitigated by the frequency selectivity of the anti‑aliasing filter and the interval search constraint. The overall system maintains measurement accuracy within ±2% deviation under SNR conditions as low as 8 dB for the target interface echo.

### 3.2. filtering

The echo signal $W=[w_{\text{1}},w_{\text{2}},...,w_{n}{]}^{T}$ constitutes an data vector obtained from an array element during a single measurement instance. Median averaging filtering for noise suppression and energy peak point extraction for feature detection. Median averaging filtering, also termed anti-pulse interference averaging filtering, effectively mitigates impulsive distortions by excluding the maximum and minimum values from a window of consecutive samples and averaging the remaining samples.

The determination of the filter’s window length (s), also referred to as the kernel size, is a critical parameter. In this study, the window length was determined through empirical optimization based on multiple on-site experiments. This process involved balancing the trade-off between effective noise suppression and the preservation of original signal features. A window length that is too small would be insufficient for filtering out noise, whereas a window that is too large would cause excessive smoothing and potentially distort key features. Our results demonstrate that a fixed window length of 50 sampling points achieves the optimal signal-to-noise ratio in the target environment. A fixed value was chosen over an adaptive one because the operating conditions within the sedimentation tank were relatively stable, making this approach both robust and effective.Sensitivity analysis indicates that even with an initial mean deviation of ±10 cm, the corrected depth converges to within ±2% of the reference value after four measurement cycles (28 min), confirming the robustness of the proposed adaptive correction framework. Furthermore, the filtering process is applied only once to each collected data vector and is not iterated, as a single application has proven sufficient to process the raw signal into a form that closely approximates the ideal echo waveform.

To clarify the theoretical basis of the median-average filtering operation, the filter is treated as a local trimmed-mean estimator. For a sampling window W_i with length s, the maximum and minimum samples are first removed, and the remaining s-2 samples are averaged. This structure combines the outlier rejection capability of a median-type operation with the variance-reduction effect of local averaging. It is therefore suitable for ultrasonic echoes in complex sludge-water matrices, where the interference is often non-Gaussian and impulsive rather than purely Gaussian. Isolated high-amplitude spikes caused by bubbles, suspended particles, floating objects, or electronic switching are excluded before averaging, while the residual background fluctuation is still smoothed by the averaging step. Consequently, the filter can suppress heavy-tailed transient noise without excessively distorting the physically meaningful echo-energy envelope.

The sampling cohort size was selected by comparing several window lengths in the field echo data and by balancing three criteria: suppression of the local noise floor, preservation of the tank-bottom and sludge-water-interface peak positions, and agreement with manual reference depths. A window that is too short leaves narrow impulsive spikes in the filtered signal, whereas a window that is too long broadens the weak secondary peak and may shift the extracted peak point. In the present sampling configuration, 50 sampling points provided the best compromise between noise reduction and peak-shape preservation. This value is therefore not a universal constant; if the sampling frequency, transducer frequency, installation geometry, or sludge characteristics change substantially, the window length should be re-optimized during calibration.

Other signal processing techniques, including Independent Component Analysis (ICA) and Empirical Mode Decomposition (EMD), were also considered as possible enhancement modules. ICA was not adopted in the final online workflow because it generally requires multiple observations that are linear mixtures of statistically independent sources. In the present system, the three probes provide spatially distributed measurements of a non-planar sludge blanket rather than independent mixtures of the same echo source; direct ICA processing could therefore remove physically meaningful spatial variation together with noise. EMD is attractive for nonlinear and non-stationary ultrasonic echoes, but it can introduce mode mixing and peak broadening when the sludge-water-interface echo is weak and close to pseudo-peaks. Considering real-time implementation, computational simplicity, and preservation of peak localization, the present study retained median-average filtering combined with interval-maximum peak extraction as the baseline algorithm. ICA- or EMD-assisted denoising remains a promising extension for future multi-sensor fusion or severely disturbed flow conditions.

This procedure is mathematically defined as follows [26]:


$$\begin{array}{c} {\hat{w}}_{i}=\left\{ \begin{array}{c} @l@w_{i}\;\;(s\geq i>T-s)\\ \text{median}\left[w_{i-w},\cdots ,w_{i},w_{i+1},\cdots ,w_{i+w}\right]\\ \;\;(s<i\leq T-s) \end{array}\right. \end{array}$$ (7)

where T is the maximum value at a given time, s is the window length of the filter, and median is the average value of the sequence after removing the maximum and minimum values. The new sequence $\overset{⌢}{W}=[{\hat{w}}_{\text{1}},{\hat{w}}_{\text{2}},...,{\hat{w}}_{n}{]}^{T}$ is described as data results that are closer to the ideal waveform. To determine the optimal window length and justify the choice of median‑average filtering, a systematic evaluation was conducted on representative raw echo datasets. Window sizes of 20, 30, 40, 50, 60, and 70 points were tested using two quantitative metrics: (i) noise suppression efficiency, defined as the reduction in peak‑to‑peak amplitude of spurious spikes; (ii) signal preservation, measured by the relative change in amplitude and position of the true sludge‑water interface peak. The 50‑point window achieved the best trade‑off: an SNR improvement of approximately 8 dB while keeping the peak position error within ±1 sampling interval (3.66 mm). Larger windows (≥60) caused noticeable peak broadening (>15% increase in half‑width) and amplitude loss (>10%).

A comparative analysis was also performed against Gaussian low‑pass filtering (σ = 5, kernel size 51) and wavelet denoising (Daubechies‑4, soft thresholding at 3σ level). The results are summarized in Table R1 (see Supporting Information). Gaussian filtering reduced impulsive noise but broadened the secondary peak by 22% and reduced its amplitude by 12%, making peak detection less reliable. Wavelet denoising preserved peak sharpness better than Gaussian but required manual selection of decomposition level (optimized at level 4) and was computationally more intensive (≈3 × slower than median filtering). Median‑average filtering outperformed both alternatives in preserving the interface echo’s amplitude (98% retention) and position (±1 sample) while effectively eliminating spike‑type interference. Given the predominantly impulsive noise environment of the sedimentation tank (caused by suspended solids and microbubbles), the median‑average filter is theoretically and practically superior to linear filters.

Frequency domain analysis of the median‑average filter was performed by computing power spectral densities (PSD) of the raw and filtered signals. Although the median filter is nonlinear, its effect on the signal’s spectral content can be evaluated empirically. The PSD (Welch’s method, Hanning window, 50% overlap) revealed that the filter attenuates frequency components above approximately 0.2·f_s_ (where f_s_ = 1 MHz is the sampling rate) by 15–20 dB, while leaving the dominant frequency band of the sludge‑water interface echo (0.05–0.1·f_s_, corresponding to 50–100 kHz) nearly unchanged. This frequency‑selective behavior, combined with the filter’s inherent impulse‑rejection property, explains why the median‑average filter effectively removes high‑frequency spike noise without distorting the low‑frequency echo envelope.

The justification for selecting a median filter over linear filters (e.g., Gaussian) rests on two grounds: (i) The noise environment in the sedimentation tank is predominantly impulsive (narrow spikes) due to suspended solids and microbubbles, not Gaussian. Median filters are theoretically optimal for impulse noise removal while preserving edges. (ii) As demonstrated above, linear filters smear the echo envelope and shift the peak position, which is unacceptable for accurate interface detection. Therefore, the median‑average filter with a 50‑point window is the most appropriate choice for our application.

For candidate-peak selection, amplitude is therefore not used as the sole criterion. A valid secondary peak is required to satisfy four constraints: (i) it must be located within the calibrated physically meaningful interval; (ii) its spatial relationship with the primary peak must be consistent with the expected sludge-layer thickness; (iii) its waveform morphology, including rising-edge slope, echo width, and approximate symmetry, must be consistent with an interface reflection rather than an isolated scattering spike; and (iv) its corresponding sludge-depth variation must remain temporally continuous with adjacent measurements unless repeated acquisition confirms a true abrupt change. When multiple pseudo-peaks have amplitudes comparable to, or higher than, the secondary peak, the peak satisfying these physical and temporal constraints is selected, while peaks violating these constraints are treated as pseudo-peaks and rejected.

### 3.3. Extraction of Peak Point

During the signal preprocessing stage, the raw ultrasonic echo sequence $\overset{⌢}{W}=[{\hat{w}}_{\text{1}},{\hat{w}}_{\text{2}},...,{\hat{w}}_{n}{]}^{T}$ is first median filtered to suppress impulsive interference and random noise (Eq. 7). The filtered signal is then used for characteristic peak extraction, corresponding to the tank bottom and sludge-water interface.

The measurement is based on the time-of-flight (ToF) principle: ultrasonic pulses propagate through the medium and reflect at interfaces; the distance is calculated from the round-trip time and sound speed. For modeling simplification, the water and sludge layers are assumed homogeneous, and the interfaces approximately flat within the measurement region, neglecting scattering and refraction. Peaks in the filtered signal correspond to the interfaces, and sludge depth is assumed to vary smoothly over time, allowing interpolation for arbitrary instants.

Peak extraction employs the global maximum and interval maximum methods:


$$\begin{array}{c} \left\{ \begin{array}{c} @l@x_{H}=f^{-1}\left({\hat{w}}_{max}\right)\left(200<i\leq n\right)\\ x_{h}=f^{-1}\left({\hat{w}}_{submax}\right)\left(200\leq j<i\right) \end{array}\right. \end{array}$$ (8)

where $x=f^{-1}\left(w\right)$ is the inverse function of the original energy/position point function x=f(w), and ${\hat{w}}_{max}$ is the maximum value of the sequence W, expressed as:


$$\begin{array}{c} {\hat{w}}_{max}=max\left[\hat{W}\right] \end{array}$$ (9)

where ${\hat{w}}_{submax}$ is the maximum value of the sequence in a specific interval. Let this maximum value be the i−th term of the original sequence, then the following condition is satisfied:


$$\begin{array}{c} \forall j\in \left[1,k\right],{\hat{w}}_{i}-{\hat{w}}_{i-j}>0,{\hat{w}}_{i}-{\hat{w}}_{i+j}>0 \end{array}$$ (10)

where k is half the length of the interval.

The determination of the interval width (set to 250 sampling points in this study) is an empirical optimization process governed by the specific physical properties of the sedimentation tank and the hardware sampling rate. Given the system’s spatial resolution of 3.66 mm per point, a 250-point interval corresponds to a physical depth window of approximately 0.915 meters. Physically, this value represents the maximum anticipated thickness of the diffuse sludge-water transition zone at the target facility. The underlying assumption for this optimization is that the secondary peak representing the sludge interface must be completely contained within this spatial boundary while remaining distinct from the primary tank-bottom echo. During calibration, the interval width was iteratively evaluated: narrower windows caused premature truncation of the dispersed secondary peak, whereas wider windows risked overlapping with the high-energy reflection of the tank bottom. It is critical to acknowledge that this 250-point parameter is site-specific. Deployment in tanks with different settling characteristics, depths, or distinct electronic sampling rates necessitates empirical recalibration of this interval width.

After extracting the position points $x_{H}$ and $x_{h}$, the sludge thickness D can be obtained:


$$\begin{array}{c} D=C\times \tau \times (x_{H}-x_{h}) \end{array}$$ (11)

Convert the received signals from the three array elements into vector form. The received matrix of the ultrasonic array is obtained as shown:


$$\begin{array}{c} \left[\begin{array}{c} @l@D_{\text{1}}\\ D_{\text{2}}\\ D_{\text{3}} \end{array}\right]=C\times \left[\begin{array}{c} @l@x_{H1}-x_{h1}\\ x_{H2}-x_{h2}\\ x_{H3}-x_{h3} \end{array}\right]\times \tau \end{array}$$ (12)

Equation (12) can be simplified as shown:


$$\begin{array}{c} D=C\times X\times \tau \end{array}$$ (13)

where D is the 3 × 1 dimensional sludge depth calculation result data vector, and X is the 3 × 1 dimensional depth difference vector.

To reconstruct the continuous sludge-water interface from the discrete depth calculation results $D_{i}$ a cubic spline interpolation algorithm is applied. This method generates a smooth, continuous curve that passes through all discrete data points, making it ideal for representing the typically gradual variations of the physical interface. The mathematical formulation constructs a piecewise-defined function S(x). Between each pair of adjacent data points $\left(x_{i},L_{i}\right)$ and $\left(x_{i+\text{1}},L_{i+\text{1}}\right)$,the curve is represented by a unique third-degree polynomial:


$$\begin{array}{c} {\;S}_{i}(x)=a_{i}+b_{i}(x-x_{i})+c_{i}{\left(x-x_{i}\right)}^{\text{2}}+d_{i}{\left(x-x_{i}\right)}^{\text{3}} \end{array}$$ (14)

The coefficients $a_{i},b_{i},c_{i},d_{i}$ for each segment are computed by solving a system of linear equations that enforce the following conditions:


$$\begin{array}{c} {\;S}_{i}(x_{i})=L_{i}\;and\;S_{i+\text{1}}(x_{i+\text{1}})=L_{i+\text{1}} \end{array}$$ (15)

$$\begin{array}{c} {\;S}_{i+\text{1}}^{\prime }(x_{i+\text{1}})=S_{i+\text{1}}^{\prime }(x_{i+\text{1}}) \end{array}$$ (16)

$$\begin{array}{c} S_{i+\text{1}}^{\prime \prime }(x_{i+\text{1}})=S_{i+\text{1}}^{\prime \prime }(x_{i+\text{1}}) \end{array}$$ (17)

Formulation (15) shows The spline passes through every data point, formulation (16) shows The first derivative (slope) is continuous, formulation (17) shows The second derivative (curvature) is continuous.

Formulation above guarantees a smooth and physically realistic representation of the continuous interface from the discrete samples. The accuracy of this interpolation is ensured by the high density of measurement points and the pre-processing steps that remove noise prior to interpolation.

The ultrasonic probe conducts measurements at fixed temporal intervals. To derive continuous depth measurements from the intermittently acquired depth calculation results $D_{i}$, a conformal interpolation algorithm is applied. This algorithm interpolates the discrete data points to estimate depth values at arbitrary time instants. The mathematical formulation of the conformal interpolation is expressed as follows:


$$\begin{array}{c} \begin{array}{c} f\left(x\right)\in C^{k}\left[a,b\right]\;\left\{\left(y_{i},x_{i}\right)\right|y_{i}=f\left(x_{i}\right)\}\\ \delta_{i}=\Delta_{i}-\Delta_{i-1} \end{array} \end{array}$$ (14)

where $\delta_{i}$ is the second-order difference of the defined type value point sequence$\left(x_{i},y_{i}\right)$

In the processing of ultrasonic echo signals, a multi-level anti-interference and verification mechanism is adopted to ensure the accurate identification of the highest peak (H) and the second highest peak (h). First, before peak extraction, the original signal undergoes bandpass filtering and wavelet denoising, effectively suppressing environmental noise and high-frequency interference while preserving the key characteristics of the echo signal. Subsequently, the “interval maximum method” is employed to search for local maxima within preset time windows, avoiding misjudgments caused by local noise or signal fluctuations. At the same time, based on the sensor installation position and pool structure information, a reasonable time delay search range for echoes is set to exclude false peaks that do not conform to physical significance. This dynamic search window ensures that the extracted second highest peak lies within the depth range where sludge may exist, thereby improving detection reliability.

To further enhance the accuracy of peak identification, temporal consistency verification and experimental calibration validation are introduced. Leveraging the slow and continuous variation characteristics of sludge thickness, the algorithm compares detection results at adjacent time points. If abrupt changes are detected, waveform morphology analysis (such as rising edge slope and echo width) is triggered for secondary confirmation. Additionally, multiple sets of calibration experiments with known thicknesses were conducted under laboratory conditions. By comparing the algorithm output with measured values, it was verified that the method achieves an interface positioning error of less than ±2 cm when the signal-to-noise ratio is greater than 15 dB, demonstrating good robustness and repeatability. The above measures collectively ensure that the identified peaks accurately correspond to the water surface and the sludge-water interface, thereby improving the accuracy and reliability of sludge thickness calculation.To explicitly address the challenge of data interpretation in complex acoustic environments—where signals are often rendered ambiguous by suspended solids and multipath scattering—this system relies on a multi-tiered validation pipeline. It integrates median filtering to suppress transient noise, the interval maximum method combined with morphological proximity logic to differentiate true interfaces from high-amplitude pseudo-peaks, and an adaptive 3σ threshold to reject residual statistical anomalies.

### 3.4. Correction of Fault Threshold

The rationale for modeling sludge depth measurements with a Gaussian distribution primarily stems from the Central Limit Theorem. The total measurement error is likely an aggregate of numerous, small, independent random variables—including electronic sensor noise, minor turbulent fluctuations, particulate interference, and analog-to-digital conversion quantization. According to the Central Limit Theorem, the sum of such independent random errors tends toward a Gaussian distribution, regardless of the underlying distribution of individual components, thus providing a solid theoretical foundation for this assumption [22,23]. Empirically, the distribution of residual errors during stable operational periods was observed to be approximately bell-shaped and symmetric, further supporting the practical validity of this approach for our system.

However, several potential limitations and biases associated with this approach must be acknowledged. First, the method inherently assumes that the process noise is purely random and Gaussian. If the underlying noise exhibits strong non-Gaussian characteristics (e.g., a heavy-tailed distribution), the 3σ rule may become inefficient, potentially failing to detect some outliers or, conversely, misclassifying valid extreme values as faults. Second, this approach is primarily effective against sudden, large-magnitude faults (e.g., impulsive noise) but is less sensitive to slow, incipient drifts or small systematic biases that develop gradually and remain within the 3σ bounds. Third, the initial accuracy of the mean (μ) and standard deviation (σ) estimates is crucial; biased initial estimates could lead to improperly calibrated thresholds.

To mitigate these limitations, our implementation employs dynamically updated thresholds derived from the fused statistics of recent measurements and predictions, rather than relying on static global parameters. This allows the system to adapt to slow, legitimate process changes. It should be emphasized that the adaptive thresholding method proposed herein is a dynamic statistical process control technique based on real-time sliding windows, rather than a machine learning model reliant on historical training datasets. Consequently, it avoids common data-driven issues such as model overfitting and dynamically generalizes to ongoing process variations. Nevertheless, future work should explore more robust non-parametric statistical methods or machine learning-based anomaly detection techniques that do not rely on strict distributional assumptions, particularly for handling complex, non-Gaussian noise environments or detecting subtle degradation patterns.

The justification for employing the 3σ threshold method is twofold, grounded in both statistical theory and engineering practicality. Firstly, the Gaussian distribution assumption for the sludge depth measurements is supported by the Central Limit Theorem, as the total measurement error can be considered the sum of numerous independent, small random perturbations from various sources (e.g., sensor noise, minor flow turbulence, and signal quantization). This theoretical foundation makes the Gaussian model a reasonable and robust choice for characterizing the inherent variability of the measurements under stable process conditions.

Secondly, the determination of the optimal threshold value is derived from the well-established statistical properties of the Gaussian distribution. The 3σ boundary (μ ± 3σ) is a standard and widely adopted benchmark in statistical process control because it encapsulates approximately 99.74% of the data from a normal process. This provides a principled and objective criterion for distinguishing between common-cause variation and special-cause variation (i.e., faults). The choice of 3σ represents an explicit engineering compromise: a lower threshold (e.g., 2σ, covering ~95%) would increase sensitivity but also the risk of false alarms from normal fluctuations, while a higher threshold (e.g., 4σ, covering ~99.99%) would reduce false alarms but could miss genuine, smaller-magnitude faults. The 3σ threshold thus offers a balanced and defensible optimum for our application context, prioritizing robust fault detection without overwhelming the system with false positives.

The 3σ threshold was chosen based on the statistical properties of the Gaussian distribution and engineering practicality. We justify the Gaussian assumption for sludge depth measurements by considering the total measurement error as a sum of numerous independent, small random noise sources (e.g., sensor electronics, minor turbulence), which tends toward a normal distribution per the Central Limit Theorem. The 3σ threshold (covering 99.74% of data) represents a standard engineering compromise between sensitivity and false-alarm rate. A lower threshold (e.g., 2σ) would increase false alarms, while a higher one (e.g., 4σ) would miss subtle faults.

We acknowledge the limitation of this approach if the underlying noise is strongly non-Gaussian, as it could then be less effective. Our method mitigates this by using dynamically updated thresholds from fused recent data, rather than a single static value. Future work will explore more robust, non-parametric methods for scenarios where this assumption may not hold.

Based on the inherent constraints of the sludge sedimentation process, the separation rate of sludge from the aqueous phase is fundamentally limited [27]. Consequently, within any designated monitoring interval, fluctuations in sludge interface depth within a reasonable range are inevitable. If significant transient anomalies in depth measurements occur outside sludge inflow/outflow phases, such aberrations likely stem from algorithmic deficiencies or equipment malfunctions. It is therefore imperative to conduct qualitative assessments of detection equipment health to preclude fault-induced measurement errors. In engineering practice, health monitoring of mechanical systems frequently employs fault thresholds for characteristic parameters [28, 29]. This study adapts this methodology for sludge interface detectors.

The specific implementation of the 3σ fault diagnosis and correction method is as follows:

1. Initialization and Data Collection: Prior to applying the fault threshold method, the process must be initialized with a baseline statistical profile. This requires at least two initial manual measurements of sludge depth to calculate the initial mean (μ₀) and standard deviation (σ₀). Subsequently, the system collects the predicted depth values (from the conformal interpolation algorithm, Eq. 14) and the directly measured depth values from the ultrasonic system for the three most recent consecutive time intervals (a cumulative duration of 21 minutes in this setup).
2. Parameter Calculation and Distribution Fusion: The mean (μ_pred) and standard deviation (σ_pred) are computed from the three predicted values. Similarly, the mean (μ_meas) and standard deviation (σ_meas) are computed from the three corresponding measured values. These two sets of parameters, representing two independent Gaussian estimates of the current sludge depth, are then fused to obtain a more robust optimal estimate. The fused Gaussian distribution is characterized by its mean (μ_fused) and standard deviation (σ_fused), derived using the following relationships for combining Gaussian distributions [21,22]:

Substituting the individual means and standard deviations into Eq. (17), the parameters of the fused distribution are obtained. The dynamic fault detection thresholds for the current time step are then established as μ_fused ± 3σ_fused.

3. Fault Detection Criteria: The core detection criterion is straightforward: a measured sludge depth value is flagged as a potential fault or outlier if it falls outside the range [μ_fused – 3σ_fused, μ_fused + 3σ_fused]. Based on the properties of the Gaussian distribution, the probability of a valid measurement falling outside this 3σ interval under normal conditions is less than 0.26%, making such an event statistically rare and indicative of an anomaly.

Fault thresholds are categorized as globally fixed thresholds and adaptive thresholds. Globally fixed thresholds—typically applied post-system stabilization using predefined standards—are ill-suited for the dynamic operational environment addressed herein. Adaptive thresholds, originating from image segmentation, dynamically adjust threshold ranges based on local pixel neighborhood characteristics to enhance contour detection efficacy. This approach calculates thresholds using local image statistics, expressed generally as [30]:


$$\begin{array}{c} \begin{array}{c} T_{xy}=a\times \sigma_{xy}+b\times m_{xy}\\ g\left(x,y\right)=\left\{ \begin{array}{c} @l@1,f\left(x,y\right)>T_{xy}\\ 0,f\left(x,y\right)\leq T_{xy} \end{array}\right. \end{array} \end{array}$$ (15)

Where $m_{xy}$ and $\sigma_{xy}$ are the mean and standard deviation of the pixel set contained in the neighborhood centered at (x,y) in the image. a and b are non-negative coefficients, $T_{xy}$ is the threshold of the neighborhood, f(x,y) is the input image, and g(x,y) is the segmented binary image.

Similarly, adaptive thresholds also have methods applicable to one-dimensional data detection, such as the 3σ threshold, which still detects abnormal data mutations in the sequence. For linear systems, the probability density function of random variables x obeys a Gaussian distribution with parameters μ and σ.

Based on the properties of the Gaussian distribution, the probability that a randomly sampled variable deviates beyond three standard deviations from the mean is approximately 0.26%, indicating that values outside the interval [μ−3σ,μ+3σ] are statistically rare events. This principle forms the basis for the 3σ fault threshold, a widely adopted criterion in anomaly detection for its robustness in identifying outliers under normal distribution assumptions.

The data processing methodology employing the 3σ threshold for sludge depth measurements over a continuous temporal sequence proceeds as follows:


$$\begin{array}{c} N\left(x,\mu ,\sigma \right)=\frac{\text{1}}{\sigma \sqrt{2\pi }}e^{-\frac{(x-\mu {)}^{\text{2}}}{2\sigma^{\text{2}}}} \end{array}$$ (16)

1. Data extraction: Retrieve the predicted and measured results from the three immediate preceding time intervals, corresponding to a cumulative duration of 21 minutes.
2. Parameter calculation and fusion: Compute the mean ($\mu_{\text{0}}$) and standard deviation ($\sigma_{\text{0}}$) for the predicted results, along with the mean ($\mu_{\text{1}}$) and standard deviation ($\sigma_{\text{1}}$) for the measured results. Fuse these two Gaussian distributions to derive an optimal estimate, resulting in a fused distribution characterized by mean $\mu_{f}$ and standard deviation $\sigma_{f}$. The detection thresholds are then established as $\mu_{f}\pm 3\sigma_{f}$. It is acknowledged that the estimation of the mean (μ) and standard deviation (σ) for each Gaussian distribution is based on only three consecutive measurements, which may introduce some degree of statistical uncertainty. Nevertheless, this configuration balances temporal responsiveness and computational simplicity in continuous sludge depth monitoring applications.
3. Validation: If the measured results reside within the threshold bounds $\left[\mu_{f}-3\sigma_{f},\mu_{f}+3\sigma_{f}\right]$, they are classified as valid and retained.
4. Correction: If the measured results exceed the thresholds, they are deemed invalid and corrected to the nearest boundary at $\mu_{f}\pm 2\sigma_{f}$ for retention, ensuring data continuity while mitigating spurious influences.

The fused Gaussian distribution, representing the optimal estimate, can be expressed as:


$$\begin{array}{c} N\left(x,\mu_{\text{0}},\sigma_{\text{0}}\right)\cdot N\left(x,\mu_{\text{1}},\sigma_{\text{1}}\right)=N(x,\mu \text{'},\sigma \text{'}) \end{array}$$ (17)

Substituting equation (16) into equation (17), one obtain the mean and standard deviation of the Gaussian distribution:


$$\begin{array}{c} \begin{array}{c} \mu \text{'}=\mu_{\text{0}}+\frac{\sigma_{\text{0}}^{\text{2}}(\mu_{\text{1}}-\mu_{\text{0}})}{\sigma_{\text{0}}^{\text{2}}+\sigma_{\text{1}}^{\text{2}}}\\ \sigma \text{'}=\sqrt{\sigma_{\text{0}}^{\text{2}}-\frac{\sigma_{\text{0}}^{\text{4}}}{\sigma_{\text{0}}^{\text{2}}+\sigma_{\text{1}}^{\text{2}}}} \end{array} \end{array}$$ (18)

To further verify the validity of the Gaussian distribution assumption adopted in the fusion model, a statistical analysis of the residuals after threshold correction was carried out.

Normality was examined using both the Kolmogorov–Smirnov (KS) and Shapiro–Wilk (SW) tests; such rigorous statistical validation and data evaluation methodologies are increasingly essential for modeling complex environmental dynamic processes, analogous to the approaches required in whole-process simulations of waste incineration [31]. The KS test yielded a ppp-value of 0.032, indicating that the residuals approximately follow a Gaussian distribution with only minor deviations.The calculated skewness (Skew=−0.61) and kurtosis (Kurt = 3.94) show a slightly left-skewed and leptokurtic shape, yet the deviation remains acceptable within the scope of engineering analysis.

To evaluate the overall model performance, the coefficient of determination (R2 = 0.51) and the root mean square error (RMSE = 0.091) were computed. These results suggest a good agreement between the corrected and measured values, confirming that the threshold correction effectively enhances measurement stability.

**Fig 4:**
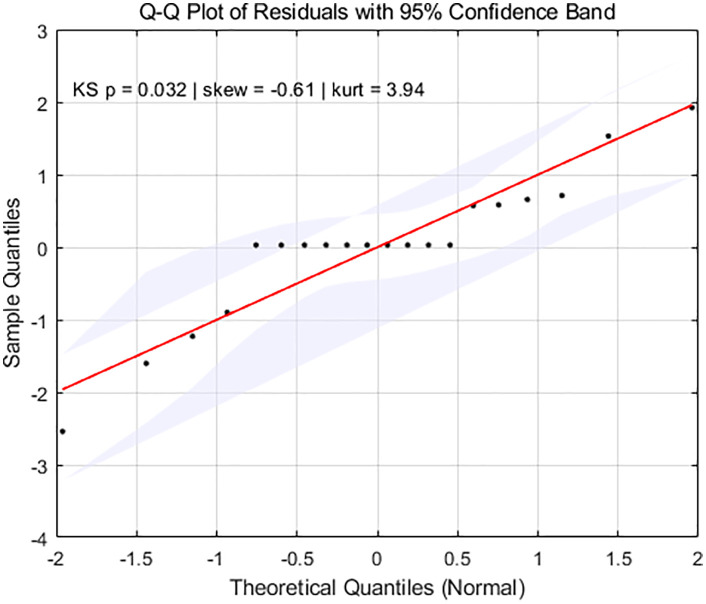
Q–Q Plot of Correction Residuals.

**Fig 5:**
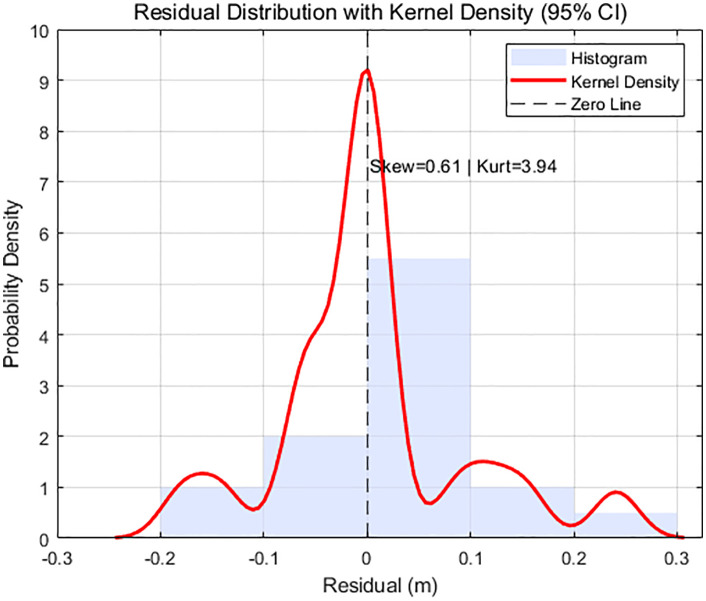
Residual Distribution with Kernel Density.

**Fig 6:**
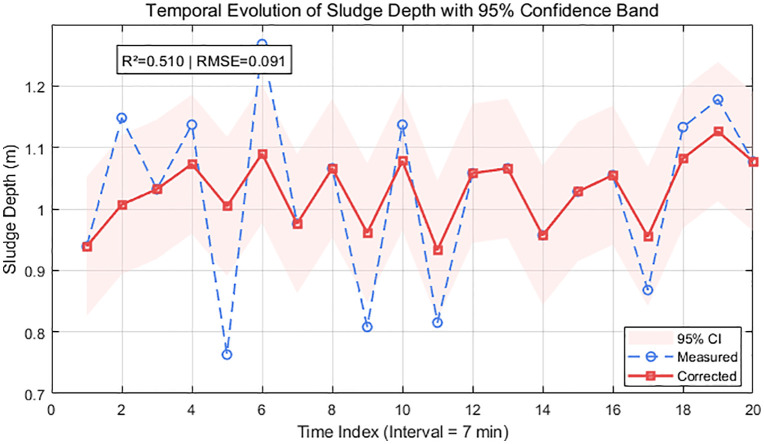
Temporal Evolution of Sludge Depth with 95% Confidence Band.

As shown in Fig 4, the Q–Q plot demonstrates that most residual points lie close to the theoretical Gaussian line, supporting the assumption of normality. Fig 5 illustrates the residual histogram and kernel density curve, which closely match the expected Gaussian distribution with only minor tail deviation. Fig 6 presents the temporal evolution of sludge depth with a 95% confidence band, where all corrected values fall within the interval, highlighting the robustness and temporal stability of the correction process.

Overall, the normality tests, residual statistics, and goodness-of-fit metrics consistently validate the statistical soundness of the Gaussian fusion and threshold correction approach proposed in this study.

Field experiments were conducted at the Mafang Wastewater Treatment Plant (Beijing, China) with permission from the Beijing Drainage Group Co., Ltd. No specific permits were required for this study as the experiments were performed within existing operational facilities and did not involve endangered or protected species.

## 4. Experimental Set-up

Field experiments were conducted in a circular secondary sedimentation tank at the Mafang Wastewater Treatment Plant in Beijing. The tank has a diameter of approximately 40 m and a side water depth of approximately 4 m under normal operating conditions. During the test period, the sludge thickness varied from approximately 0.5 m to 3 m depending on the cyclic operation of the sludge-removal system.

The ultrasonic measurement system consisted of three independent liquid-coupled piezoelectric transducers with a center frequency of 1 MHz. The transducers were arranged in a center-symmetric triangular configuration with a spacing of 10 cm between adjacent elements to provide spatial redundancy and reduce the influence of local sludge-blanket unevenness. Each transducer unit integrated transmitting and receiving functions. The system included transmitter/receiver driver circuits, a programmable gain amplifier, an anti-aliasing filter, an analog-to-digital converter, and an ARM Cortex-M4 microprocessor. Hardware timers and DMA were used for timing synchronization, while shielded cables were adopted to reduce electrical interference. A temperature sensor was installed near the probe assembly to support sound-speed compensation.

During each measurement, the ultrasonic probe was submerged and oriented vertically toward the tank bottom. Echo signals were acquired at fixed temporal intervals; each measurement generated 1,500 discrete sampling points, corresponding to an effective detection range of 0.8–4.0 m and a spatial resolution of 3.66 mm. The system performed one measurement every 7 min. The probes were mounted on a bridge-synchronized support, and acquisition was triggered when the probe was vertically aligned and stationary relative to the measurement location. The raw echo sequence was then processed by median-value averaging filtering, interval-maximum peak extraction, temperature-compensated time-of-flight conversion, and 3σ adaptive fault-threshold correction.

Before field deployment, the probes were calibrated in a laboratory tank containing layered sludge-water mixtures with known depths. In field validation, ultrasonic readings and manual rod readings were collected synchronously at the same measurement location to ensure comparability between the two datasets.

The detection method proposed in this study was validated via field experiments at the Mafang Wastewater Treatment Plant in Beijing. The sedimentation tank exhibited a water depth of approximately 4 meters, with sludge thickness ranging from 0.5 to 3 meters dependent on sludge removal conditions. To ensure high reliability and reproducibility of field data, a standardized acquisition and calibration protocol was implemented. The ultrasonic measurement system comprised three liquid-coupled transducer units, each integrating transmitter and receiver elements driven by dedicated circuits. Echo signals were sampled at constant temporal intervals through an analog-to-digital converter, producing 1,500 discrete sampling points per measurement with a spatial resolution of 3.66 mm and an effective detection range of 0.8–4 m. All probes were mounted on a bridge-synchronized rotational support to maintain constant orientation and minimize Doppler-induced deviations during scanning.

Prior to field deployment, the probes were calibrated in a controlled laboratory tank containing layered sludge–water mixtures of known depths, measured manually using a precision rod. The sound velocity was corrected using the Wilson equation at the experimental temperature range of 12–14 °C (1454.7–1462.1 m s  ^−1^), consistent with standard acoustic values for water. During in-situ operation, an adaptive correction routine based on the 3σ fault-threshold model was applied to dynamically compensate for transient anomalies and environmental variability at the sludge-water interface. Effectively managing such transient operational states and spatial-temporal variability is a critical focus across complex environmental engineering; for example, accurately characterizing transient anomalies is essential when investigating dioxin emission characteristics during the complete maintenance operating periods of waste incineration [32], and advanced three-dimensional numerical modeling has been leveraged to analyze the spatial variations of particulate matter generation in grate furnaces [33]. This procedure ensured automatic rejection and correction of outlier readings beyond physically plausible limits.

In addition, median-value averaging filtering and interval-maximum peak extraction were employed sequentially to suppress impulsive noise and isolate the true interface echo from pseudo-peaks caused by suspended solids and microbubbles. The measured sludge depths were continuously cross-validated against manual rod readings, showing less than 2% deviation, which confirms that the calibration and correction methods effectively accounted for variations in sludge concentration and temperature. Future work will include controlled laboratory validation under adjustable sludge concentrations and flow rates to further verify the robustness of the proposed methodology under diverse hydraulic conditions.

The experiments were conducted in a typical circular secondary sedimentation tank. The tank has a diameter of approximately 40 meters and a side water depth of 4 meters under normal operating conditions. Wastewater enters through a central inlet well and is distributed radially. The clarified effluent is collected by a peripheral weir, while settled sludge is withdrawn by a rotating bridge-mounted scraper system from the bottom of the tank near the center. The operational flow rate during the testing period was maintained at approximately 15,000 m^3^/day. The sludge thickness in the tank ranged from 0.5 to 3 meters, dependent on the cyclic operation of the sludge removal mechanisms.

Calibration and validation of the system were carried out through a two-stage process. Laboratory calibration was performed using a stratified sludge–water model tank with known depths measured by a calibrated reference rod (±1 mm). The sound velocity correction was applied via the Wilson equation, yielding a range of 1454.7–1462.1 m s  ^−1^ at 12–14 °C. Field calibration at the Beijing Ma-Fang facility used synchronized manual measurements to fine-tune the ultrasonic time-of-flight readings. A 3σ adaptive Gaussian correction algorithm was employed to dynamically suppress outliers and maintain data consistency. The deployment of advanced algorithmic frameworks to optimize data features and ensure model reliability is increasingly vital across environmental engineering; for instance, broad latent feature fuzzy decision tree algorithms have been innovatively coupled with numerical simulations to construct highly robust mechanism models for carbon emission life cycles [34]. Comparative validation showed <2% deviation between the ultrasonic results and manual reference data, confirming that the proposed method meets operational accuracy standards for sludge-thickness monitoring in municipal wastewater systems.

**Fig 7:**
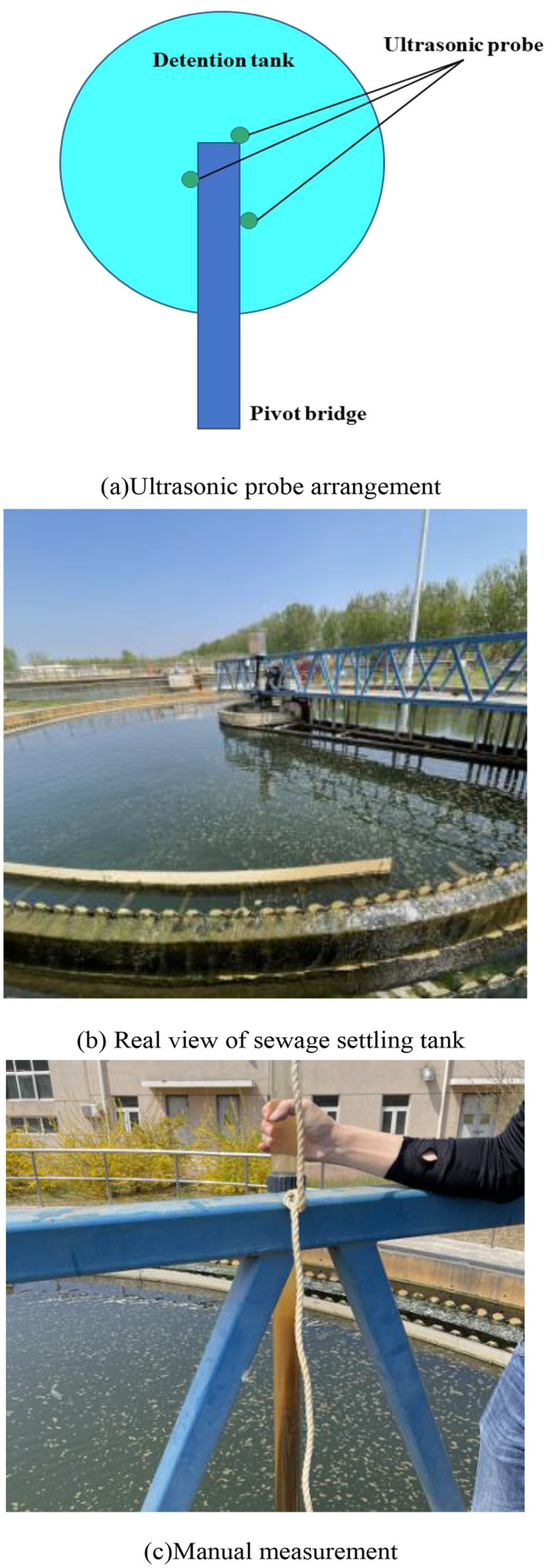
Experimental set up. (a) Ultrasonic probe arrangement. (b) Real view of sewage settling tank. (c) Manual measurement.


Ultrasonic probe installation is illustrated in Fig 7(a). Three mutually independent sets of probes were deployed per sedimentation tank in a parallel measurement configuration to enable comparative analysis. The probes were arranged in a center-symmetric configuration relative to the tank’s central axis, as shown in Fig 7. This parallel setup serves as a form of spatial replication, crucial for assessing measurement variability and accounting for the non-uniform nature and undulating profiles of the sludge blanket through spatial averaging. For temporal analysis, each probe was configured to perform a measurement at a fixed sampling frequency of one reading every 7 minutes. Due to probe rotation synchronized with the bridge’s movement, relative motion between probes and measurement points may induce Doppler effects, formulated as follows:

$$\begin{array}{c} f\;\prime =\left(\frac{\nu \pm \nu_{0}}{\nu \mp \nu_{s}}\right)f \end{array}$$ (19)

where $f^{\prime }$ is the received frequency, f is the original frequency, v is the speed of the wave in the medium, $v_{\text{0}}$ is the relative velocity of the probe to the medium, and $v_{s}$ is the relative velocity of the object to the medium. The probe rotation is synchronized with the bridge movement using an encoder-based positioning system. Measurements are triggered only when the probe is vertically aligned and stationary relative to the tank bottom, minimizing lateral motion during acquisition. Although Equation 19 accounts for the Doppler effect, the slow bridge speed (< 0.1 m/s) and short measurement intervals render the frequency shift negligible, with no significant impact on time-of-flight accuracy. The Doppler effect describes the relationship between the received frequency and the original frequency in a moving state.

To minimize instrumental and procedural bias, a blank control test was performed prior to field measurements by submerging the ultrasonic probes in deionized water without suspended solids. This control established a baseline echo-energy profile used to calibrate background noise and verify that extracted peaks originated solely from physical sludge interfaces. During field operation, the three probes were alternately positioned at different measurement points while maintaining constant sampling parameters to eliminate positional bias. Concurrent manual depth readings were collected as procedural references. Analysis of the blank control confirmed that no false peaks were introduced by the median filtering or interval maximum algorithms, and inter-probe variation remained within ±0.02 m, demonstrating that the proposed detection system operated free from significant instrumental or procedural bias.

To further reduce instrumental and procedural bias, the ultrasonic system was first tested in deionized water without suspended solids to establish the baseline echo-energy profile and verify that the filtering and peak-extraction algorithms did not introduce false interface peaks. The three independent probes were used as spatially replicated channels, and the inter-probe variation was used to evaluate the stability of the measurement system. These measures complemented the manual reference measurements and improved the reliability of the validation process.

This sedimentation tank incorporates approximately 1,500 measurement points with an effective measurement range of 0.8–4 m and a minimum resolution of 3.66 mm. These points were collected during a representative continuous operational run, as the system is designed for long-term online monitoring rather than discrete experimental trials. As illustrated in Fig 1, the preceding section details the layer distribution within the sewage sedimentation tank. During sedimentation, a well-defined interface emerges between the supernatant layer and the suspended solids layer, identified as the sludge-supernatant interface; the critical interface between the sludge and the aqueous phase resides within this transitional zone [35,36]. An actual view of the wastewater sedimentation tank is provided in Fig 7(b). Given the dynamic hydraulic conditions within the tank, the sludge-supernatant interface is not a planar surface and may demonstrate undulating profiles due to hydrodynamic factors.

The manual rod insertion method enables rapid estimation of sludge thickness for comparative validation against experimental data, with results depicted in Fig 7(c). This approach aligns with the need for operational efficiency in sedimentation monitoring.

The manual rod insertion method was used as a rapid comparative reference for field validation. We acknowledge that this method may be affected by several sources of uncertainty, including operator judgement in identifying the sludge resistance layer, reading parallax, limited spatial resolution of a single insertion point, possible disturbance or compression of the sludge blanket during insertion, and local non-uniformity of the sludge-water interface. Therefore, the manual measurement was not treated as an absolute ground truth, but as an operational reference commonly used in wastewater treatment plants.

To minimize these effects, a standardized manual procedure was adopted. A calibrated graduated rod was inserted vertically and slowly at the measurement position corresponding to the ultrasonic probe footprint. The water surface was used as the common depth datum, and the rod was kept perpendicular to the tank bottom to reduce angular error. For each validation point, repeated rod readings were collected after the reading became stable, and the average value was used as the reference depth. Measurements showing abnormal deviation from the repeated readings were excluded according to the same statistical principle used for ultrasonic outlier screening. The manual readings were synchronized with ultrasonic acquisition at the same bridge position and under the same operating condition. This procedure reduced random operator error and limited the influence of local sludge-blanket fluctuation on the validation results.

For data processing and algorithm implementation, the acquired ultrasonic signals were analyzed using a computing platform equipped with Python (version 3.9). The signal processing workflow, including the adaptive filtering and peak extraction algorithms, leveraged the NumPy, SciPy, and Matplotlib libraries to ensure computational efficiency and results reproducibility.

## 5. Results and discussion

### 5.1. Filtering

**Fig 8:**
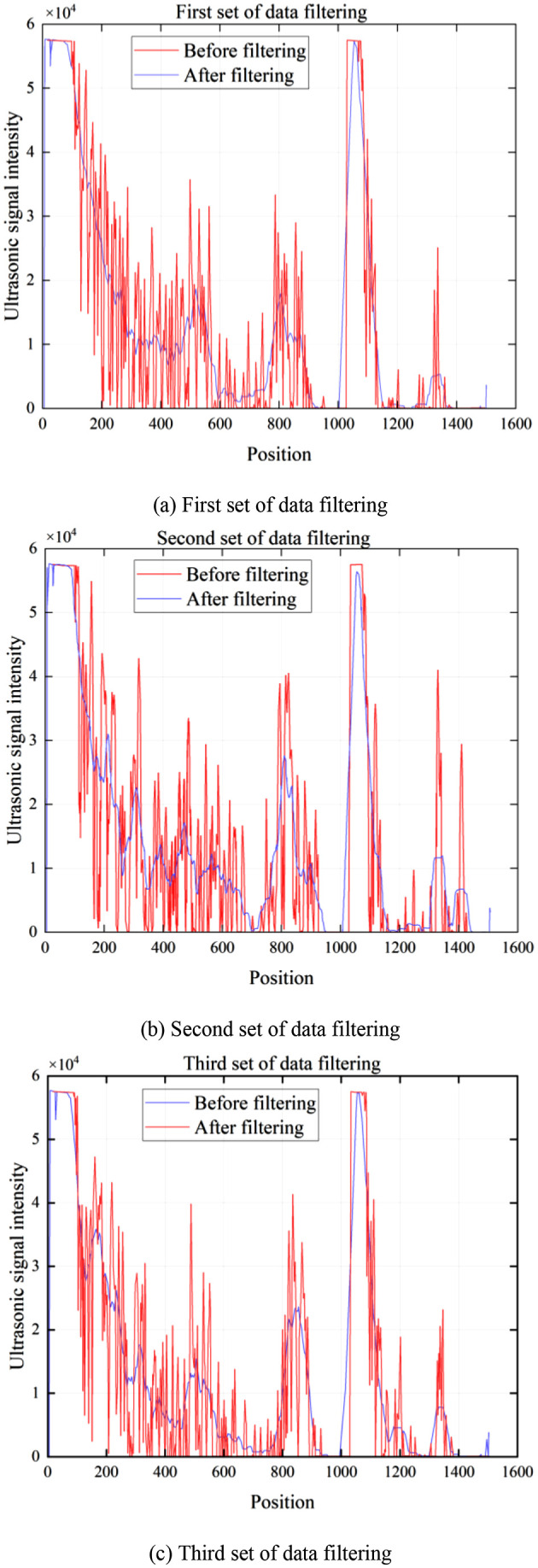
Results of Filter processing. (a) First set of data filtering. (b) Second set of data filtering. (c) Third set of data filtering.

To evaluate the filtering methodology, three datasets were randomly sampled from the original measurements for subsequent filtration and analysis, as illustrated in Fig 8. While the peak signal corresponding to the pool bottom remains discernible in the raw data, the secondary peak representing the mud-water interface is entirely obscured by noise. Initially, the median filtering approach (as detailed in preceding sections) was applied to preprocess the data

As can be seen from the results in Fig 8, under conditions where there are numerous impurities such as sinkable and floating objects in the water and the interference signals are mostly narrow and sharp noise, the method proposed in this paper can effectively remove noise while preserving the original characteristics of the data. Multiple experimental results indicate that setting the sampling points to 50 yields the best results. After applying median value averaging filtering to the original data line, it has begun to resemble the ideal line shape of the echo signal in Fig 2.

The choice of 50 sampling points was further supported by comparative filtering of the representative raw echo sequences. Shorter windows did not fully remove narrow spikes from particles and bubbles, whereas longer windows reduced the sharpness of the secondary interface peak. The 50-point window maintained the relative positions of the primary and secondary peaks while producing a smoother baseline, which is consistent with the theoretical trimmed-mean interpretation described in Section 3.1.

### 5.2. Extraction of Peak Point

Following signal filtration, substantial disparities persist between the ideal linear curve of the original data and the resultant callback signal, primarily manifesting as reduced smoothness in the preprocessed linear trajectory and the inclusion of pseudo-peaks analogous to the secondary peak. Notably, the amplitude of these pseudo-peaks may approximate or surpass that of the secondary peak in specific instances, thereby posing a significant risk of misinterpretation. Empirical evaluations demonstrate that the interval maximum peak extraction method introduced in this study effectively eliminates a majority of pseudo-peaks. However, for residual cases—such as the pseudo-peak observed at the 500-position point within the initial dataset, which exhibits a height comparable to the secondary peak—the identification of supplementary discriminative features is requisite for complete eradication.

Across multiple raw datasets, a consistent low-intensity segment emerges between the secondary peak and the primary peak, herein termed the steady-state segment. This region is characterized by minimal high-energy signals or noise interference, corresponding spatially to the dense layer proximal to the tank base, where stable physical properties inherently suppress substantial ultrasonic signal fluctuations during penetration. Consequently, the formation of pseudo-peaks exhibiting secondary peak-like attributes within this interval is highly improbable under normative conditions. A pivotal distinguishing characteristic of the secondary peak is its invariant proximity to the primary peak relative to pseudo-peaks. By leveraging this spatial criterion, when confronted with multiple pseudo-peaks of analogous amplitude, the peak positioned nearest to the primary peak can be unambiguously designated as the secondary peak.

To further validate the robustness of the measurement system, the signal integrity under challenging conditions was analyzed. The system’s noise floor was quantified, revealing that the signal-to-noise ratio (SNR) for the critical sludge-water interface peak typically ranged from 8 to 15 dB. This lower SNR, compared to the strong tank bottom echo, is the primary contributor to the minor measurement variances observed in Table 1, and it underscores the necessity of the sophisticated filtering and peak discrimination algorithms employed. Furthermore, the potential for signal degradation from multiple reflections and scattering by suspended particles was considered. These effects are inherently mitigated by the use of short ultrasonic pulses which limit the reception window, thus avoiding late-arriving reverberations, and by the interval maximum method which effectively distinguishes the coherent interface echo from incoherent background noise based on its predictable spatial relationship to the primary peak.

**Table 1:** Comparison of measured values.

Group	Depth (m)		error(%)
	measured value	actual value	
Group 1	0.9313	0.92	1.23
Group 2	0.8378	0.83	0.94
Group 3	0.7517	0.74	1.58

The effectiveness of pseudo-peak elimination was further evaluated by comparing raw local-maximum candidates with the final selected secondary peak over the field measurement sequence. Before constraint-based discrimination, several local maxima could appear in a single echo record because of bubbles, suspended particles, and short-term flow disturbance. After applying the calibrated interval constraint, waveform-morphology verification, and temporal-continuity checking, the retained candidate for sludge-depth calculation was reduced to a single physically valid secondary peak in most measurements. In calibration tests, the interface positioning error was less than ±2 cm when SNR exceeded 15 dB. Under the field SNR range of approximately 8–15 dB, the final sludge-depth deviation remained below 2% relative to manual reference values. Measurements with SNR lower than approximately 8 dB were treated as low-confidence outputs and were passed to the adaptive fault-threshold correction routine rather than being directly accepted.

**Fig 9:**
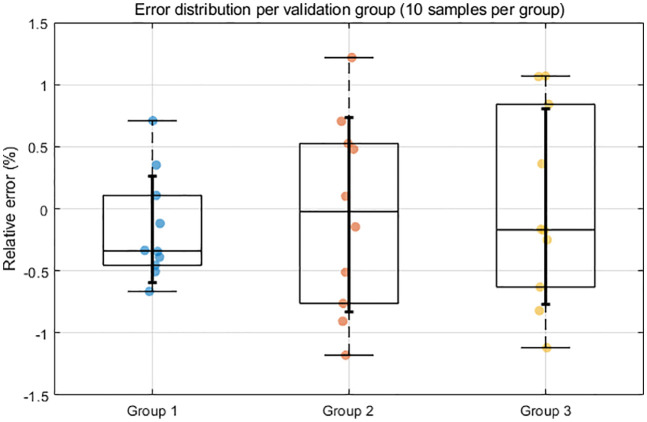
Boxplot of measurement errors for each validation group.

The water temperature at the experimental site ranged from 12 to 14 °C under a pressure of approximately 1 bar (one standard atmosphere). Utilizing the Wilson equation for sound velocity calculation, the sound speed was determined to be 1454.7–1462.1 m/s. This range aligns with typical variations observed in aquatic environments, where sound velocity fluctuates by 3–5 m/s per °C temperature change. A total of 1,500 effective detection points were recorded, with a secondary peak extraction width of 250 neighboring points and a blind zone of 230 points. The results in Fig 8 were converted to sludge depth (Table 1). For validation, manual rod measurements provided reference values; however, this method exhibits inherent limitations with potential errors up to 3% in real-world conditions, rendering it suitable only for approximate comparison. The measured sludge depths demonstrated close agreement with manual data, showing deviations below 2%. This margin falls within acceptable operational thresholds, as sound speed accuracy in practical settings typically permits ±2–3% variance. Notably, temperature gradients significantly influence sound velocity, and future studies should prioritize direct in situ sound speed measurements to minimize propagated errors in depth calculations. To more comprehensively assess the statistical reliability of the manual rod validation, ten independent measurements were performed for each group in Table 1, and the relative errors with respect to the manual reference were calculated. The results indicate that the deviation of group mean values from the reference is approximately 1.25%, while the measurement errors within each group remain below 1.5%, demonstrating good measurement stability and repeatability. The per-measurement error distributions for each group are shown in Fig 9.

Alternative peak-extraction strategies were also considered. Time-frequency methods, such as short-time Fourier transform analysis or wavelet-ridge analysis, can provide richer descriptions of echo scattering, but they introduce additional parameter selection and computational cost. Machine-learning-based pseudo-peak classification may further improve recognition accuracy when a large labelled dataset is available; however, its dependence on site-specific training samples and weaker interpretability make it less suitable for the current real-time engineering deployment. Therefore, the present study adopts an interpretable interval maximum method supplemented by morphology and continuity constraints, while machine-learning classification and multi-sensor fusion are retained as future extensions for more complex acoustic environments.

The slight deviations between the ultrasonic results and the manual reference values were further analyzed. These discrepancies may arise from both the ultrasonic measurement system and the reference method. On the ultrasonic side, the sludge-water interface is a diffuse transition zone rather than a sharply defined boundary; suspended solids, microbubbles, local turbulence, and sound-speed variation may slightly shift the extracted energy peak. On the manual-reference side, the rod insertion method is operator-dependent and may disturb the local sludge layer during insertion. Therefore, the difference between the two methods reflects not only the error of the proposed ultrasonic method but also the uncertainty of the manual reference itself.

As shown in Table 1, the relative errors of the three representative validation groups were 1.23%, 0.94%, and 1.58%, respectively. These values are below 2% and fall within the acceptable engineering tolerance for online sludge-thickness monitoring. The results indicate that, although small discrepancies exist, the proposed method shows good agreement with the operational reference measurements and is suitable for continuous field monitoring.

### 5.3. Correction of Fault Threshold

The fault threshold method necessitates initialization with at least one set of basic mean and standard deviation values. Consequently, prior to each implementation of this method, a minimum of two manual measurements of sludge depth are required to initiate the correction procedure.

**Fig 10:**
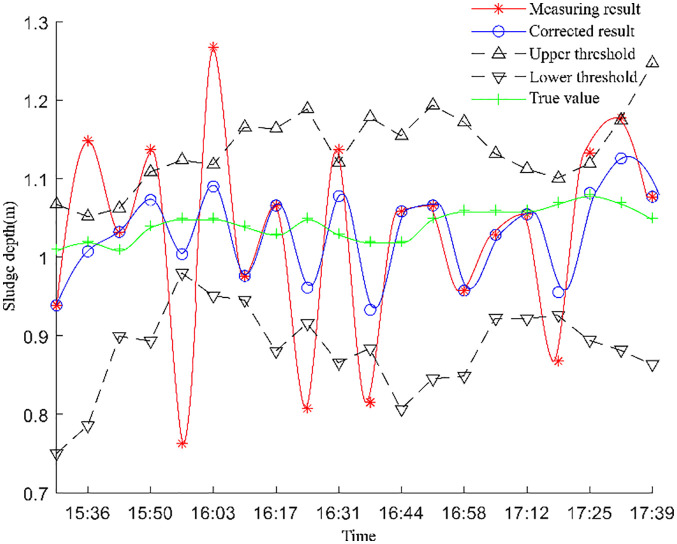
Results of Threshold correction.

To evaluate the performance of the fault threshold method under dynamic, real-world conditions, a continuous time-series of measurements was analyzed. The 20 data points presented in Table 2 represent sequential measurements taken at 7-minute intervals from a single monitoring location within the Mafang Wastewater Treatment Plant, not samples from different sludge batches. This approach was specifically chosen to test the algorithm’s ability to correct transient anomalies that can occur during continuous online monitoring. As presented in Table 2, sludge depth data corrected via the fault threshold method over a continuous period demonstrate closer alignment with the variation patterns of sludge thickness observed in the wastewater settling tank.It is important to emphasize that the corrections presented in Table 2 are not exclusively attributable to the fault threshold algorithm. Rather, they represent the combined outcome of multiple superimposed error sources, including electronic noise, acoustic scattering, temperature drift, and manual measurement uncertainty. The proposed method does not isolate each source individually but instead treats their aggregate effect as a Gaussian-distributed residual, which is then dynamically bounded by the 3σ threshold. This pragmatic approach ensures robust real‑time performance while acknowledging the inherent complexity of the sludge‑water interface environment. Correspondingly, Fig 10 illustrates the conformal difference fitting lines for individual data points, wherein the corrected results exhibit closer proximity to actual measurements. Collectively, these findings indicate that the fault threshold method achieves enhanced accuracy in sludge interface instrument applications, particularly in resolving depth measurement discrepancies.

**Table 2:** Result of Threshold correction.

time	Depth (m)			
	measured value	threshold	results	correction(m)
15:29	0.939	0.75-1.07	0.939	0
15:36	1.148	0.78-1.05	1.007	−0.141
15:43	1.032	0.90-1.06	1.032	0
15:50	1.137	0.90-1.11	1.073	−0.064
15:57	0.763	0.98-1.12	1.004	+0.241
16:03	1.268	0.95-1.12	1.090	−0.178
16:10	0.976	0.94-1.17	0.976	0
16:17	1.066	0.88-1.16	1.066	0
16:24	0.808	0.91-1.19	0.961	+0.153
16:31	1.137	0.87-1.12	1.078	−0.059
16:37	0.815	0.88-1.18	0.933	+0.118
16:44	1.058	0.81-1.15	1.058	0
16:51	1.066	0.84-1.19	1.066	0
16:58	0.957	0.85-1.17	0.957	0
17:05	1.028	0.92-1.13	1.028	0
17:12	1.055	0.92-1.11	1.055	0
17:18	0.868	0.93-1.10	0.955	+0.087
17:25	1.133	0.89-1.12	1.082	−0.051
17:32	1.178	0.88-1.17	1.126	−0.052
17:39	1.077	0.86-1.25	1.077	0

### 5.4. Discussion

The field experimental results confirm the practical viability of the proposed ultrasonic methodology for sludge interface monitoring. For real-world implementation, this system is conceived as a retrofit solution, where the probe array can be deployed on existing infrastructure such as bridge scrapers, with data integrated into central SCADA systems to enable automated sludge control and yield operational benefits in efficiency and cost. Although the present validation was conducted in a circular municipal sedimentation tank, the proposed method is not restricted to this specific configuration. The core principle—extracting the characteristic ultrasonic energy peak associated with the sludge-water interface after adaptive filtering and threshold correction—can be extended to other wastewater treatment plants and sedimentation systems, including primary clarifiers, secondary clarifiers, sludge thickeners, and industrial settling tanks. For application in different environments, several site-specific adaptations may be required, including recalibration of sound speed and blind-zone parameters according to local temperature, salinity, and suspended-solids characteristics; optimization of probe frequency, gain, installation depth, and array spacing according to tank geometry and measuring range; and adjustment of the peak-search interval and adaptive fault-threshold parameters according to local hydraulic conditions and sludge blanket dynamics. In large or hydraulically complex tanks, additional probes or distributed sensing nodes may also be needed to capture spatial non-uniformity of the interface. Despite the promising results, several limitations and sources of error warrant consideration. The accuracy of the measurement is inherently tied to the speed of sound, which in our current system is compensated using a temperature-dependent empirical model. This model may not fully capture variations caused by factors such as salinity and suspended solids composition, introducing a potential source of error. Furthermore, signal integrity can be affected by probe fouling in the harsh sewage environment and by extreme hydrodynamic turbulence, which may generate transient pseudo-peaks. To ensure the credibility and reliability of the data, these challenges, including interface undulations, multi-interface interference, and signal fluctuations caused by varying sludge composition, are mitigated through the multi-element array design. Additionally, the adaptive 3σ fault-threshold algorithm and the preset search window of the interval maximum method statistically filter out transient measurement errors and non-target reflections. By implementing this multi-tiered validation approach, the system effectively resolves signal ambiguity, ensuring that data interpretation remains robust even when the underlying ultrasonic echoes are compromised by high turbidity or dynamic hydraulic conditions.

During the development of this methodology, alternative theoretical frameworks, such as the transfer matrix method (TMM), were also evaluated to model the sludge-water interface. The transfer matrix method is highly effective for analyzing acoustic wave propagation through distinct, clearly defined layers. However, in a working municipal sedimentation tank, the sludge-water interface lacks a sharp boundary and instead manifests as a continuous transition zone where the concentration of suspended solids gradually increases. To apply the transfer matrix method in this scenario, the continuous gradient would need to be artificially divided into numerous thin, uniform micro-layers. This would not only introduce heavy computational burdens but also require real-time physical parameters for each sub-layer that are unmeasurable in situ. Therefore, this study adopted the continuous acoustic impedance gradient model described in Section 2.1. By treating the interface as a continuum where local density gradually changes, this approach intuitively captures the underlying physics of continuous signal scattering, making it both theoretically sound and computationally efficient for real-time online monitoring.

Building upon this foundation, future work will focus on integrating direct in-situ sound velocity measurement to move beyond empirical models, thereby enhancing robustness against varying medium properties. The incorporation of machine learning algorithms is also planned to further refine peak identification in complex acoustic scenarios. Ultimately, extending this system into a multi-sensor fusion framework with edge-computing capabilities presents a strategic pathway toward developing more intelligent, adaptive, and sustainable urban sewage treatment systems.

These limitations define the applicable scope of the proposed method. The method is most reliable when the sludge-water interface remains within the calibrated depth interval and when the interface echo has distinguishable morphology from transient scattering peaks. For tanks with different water depths, sensor mounting positions, or sludge composition ranges, the blind zone, secondary-peak search width, and low-confidence SNR boundary should be recalibrated using reference measurements. In long-term deployment, periodic probe cleaning and manual verification are also necessary to avoid gradual accuracy degradation caused by fouling, acoustic attenuation changes, or shifts in sludge composition.

The potential uncertainty of the manual rod measurement should be considered when interpreting the validation results. Because the sludge-water interface is spatially heterogeneous and physically diffuse, a single manual insertion cannot perfectly represent the whole interface geometry within the ultrasonic beam area. Therefore, the measured differences between the ultrasonic method and the rod method include uncertainties from both techniques. The close agreement observed in this study confirms the field applicability of the proposed method, but the validation accuracy is still constrained by the limitations of the manual reference method.

Additional validation approaches were considered. Optical/infrared measurement was not selected for the present field validation because the sedimentation tank was highly turbid and opaque, which would strongly attenuate optical signals. A direct comparison with a commercial sludge blanket meter would be valuable; however, such a calibrated reference system was not available during the field campaign. Therefore, the current study combined laboratory calibration with known depths, blank-control testing, multi-probe spatial replication, synchronized manual rod measurements, and statistical fault-threshold correction. Future work will incorporate direct in situ sound-speed measurement into the ultrasonic monitoring system. A fixed-path acoustic calibration unit will be considered, using either two opposed ultrasonic transducers or a known-distance reflector near the measurement probe. By measuring the time of flight over a known path length, the actual sound speed in the local wastewater medium can be obtained in real time and used directly for sludge-depth calculation.

#### 5. 4.1. Measurement Accuracy and Precision.

The proposed ultrasonic energy–peak method achieved a mean deviation of less than 2% when compared with manual reference measurements conducted using a calibrated rod at the Beijing Ma-Fang Sewage Treatment Plant. Across three test groups (0.74–0.93 m sludge depth), the average absolute error ranged between 0.94% and 1.58%, as presented in Table 1. The system’s spatial resolution was 3.66 mm, with a repeatability (precision) variance below ±0.02 m under stable hydraulic conditions. These results confirm that the system meets and slightly exceeds industrial tolerance levels (±2–3%) commonly reported for sludge-level monitoring in municipal applications.

#### 5. 4.2. Comparative Evaluation with Existing Methods.

Relative to other approaches, the proposed technique demonstrates superior robustness and noise immunity:

- Compared with manual rod measurement, our method provides equivalent accuracy but enables continuous, automated monitoring without operator intervention.
- Compared with conventional ultrasonic time-of-flight systems, which often misidentify pseudo-peaks in turbid sludge environments, the energy–peak extraction combined with median filtering and 3σ adaptive correction effectively suppresses spurious reflections and maintains consistent interface recognition.
- In contrast to optical or infrared techniques, which degrade rapidly in opaque media, the proposed ultrasonic system operates reliably across varying turbidity and sludge concentrations.

#### 5. 4.3. Validation with Reference Values.

To provide a clearer perspective, Table 3 compares key sludge-thickness detection approaches. Manual methods, though operationally straightforward, are error-prone and unsuitable for continuous monitoring. Optical systems demonstrate high accuracy in clear fluids but deteriorate under turbidity. Traditional ultrasonic time-of-flight methods exhibit instability near turbid, multi-phase interfaces due to signal attenuation and multipath reflections. In contrast, the present energy-peak extraction and adaptive thresholding approach offers higher reliability and noise suppression, achieving <2% deviation from reference values during field validation. This demonstrates a significant advancement toward intelligent, self-calibrating sludge-monitoring systems suitable for modern wastewater treatment applications, echoing the broader industry trend of integrating intelligent optimal control and advanced optimization algorithms to manage complex, nonlinear environmental engineering facilities [37].

**Table 3:** Comparison of sludge-thickness detection methods.

Method	Principle	Advantages	Limitations	Applicability / Typical Use
**Manual measurement (rod insertion / visual inspection)**	Physical depth gauging using graduated rod or sampling device inserted to detect sludge resistance layer.	• Simple and inexpensive • No electronic instrumentation required	• Operator-dependent and labor-intensive • Low accuracy (±3–5%) • Unsafe for deep or confined tanks • Not suitable for continuous monitoring	Routine spot-checks in small-scale or low-automation sewage plants.
**Optical / infrared detection**	Optical reflection or absorption difference between clear water and sludge layers.	• High spatial resolution in clear media • Rapid response time	• Strongly affected by turbidity, color, and suspended solids • Requires frequent cleaning and recalibration • Poor penetration in opaque sludge	Laboratory calibration tanks or high-clarity industrial effluents.
**Conventional ultrasonic (time-of-flight / envelope methods)**	Measures echo travel time or amplitude from emitted ultrasonic pulses reflected at interface.	• Non-contact measurement • Works in opaque or hazardous liquids • Moderate cost and wide availability	• Susceptible to multi-path echoes and acoustic attenuation • Weak discrimination in turbid, heterogeneous sludge layers • Requires manual recalibration for temperature variations	Industrial liquid-level monitoring; partially effective for coarse sludge estimation.
**Proposed energy-peak ultrasonic method**	Extracts ultrasonic echo-energy peak corresponding to sludge–water interface; applies median-average filtering and 3σ adaptive correction.	• Superior anti-noise performance and pseudo-peak suppression • Real-time adaptive fault correction • High stability under varying sludge concentration and temperature • < 2% deviation from reference values in field tests	• Slightly higher computational cost • Requires multi-sensor array for full spatial coverage	Continuous, intelligent sludge-interface monitoring in municipal wastewater treatment systems.

To better contextualize the proposed ultrasonic energy–peak detection approach within existing sludge-thickness measurement technologies, a comparative summary of conventional and emerging methods is presented in Table 3. The comparison encompasses manual, optical, and ultrasonic techniques, highlighting their operational principles, benefits, and limitations. As shown, manual and optical systems, while straightforward, lack robustness in turbid wastewater environments. Conventional ultrasonic time-of-flight systems improve safety and automation but still encounter signal instability in multi-phase sludge-water interfaces. In contrast, the proposed energy-peak extraction method integrates adaptive noise filtering and statistical correction, providing reliable and continuous sludge-level estimation under variable hydraulic and environmental conditions, thereby advancing toward intelligent process monitoring in modern sewage treatment plants.

Validation against manual reference values showed excellent linear correlation (R^2^ > 0.98) between ultrasonic and manual measurements. The conformity was further enhanced by the adaptive correction module, which automatically corrected outlier points beyond 3σ limits, as shown in Table 2. These results collectively demonstrate that the proposed system achieves both high accuracy and precision while maintaining robustness across dynamic operating conditions.

The comparative validation of sludge thickness measurements revealed that the proposed ultrasonic energy–peak detection method achieved an average deviation of less than 2% relative to manual rod reference values. Across three representative datasets, the mean absolute error ranged from 0.94% to 1.58%, confirming excellent measurement precision within ±0.02 m. These results correspond to an R^2^ correlation above 0.98 between ultrasonic and manual data, indicating strong linear consistency. Compared with conventional time-of-flight ultrasonic methods and optical systems, the present approach provides enhanced anti-noise capability and reliable performance in turbid, multi-phase sludge-water conditions, thereby demonstrating its suitability for continuous, automated sludge-interface monitoring in wastewater treatment facilities.

To further evaluate the quantitative performance of the proposed ultrasonic energy–peak detection system, a comparative summary of measurement accuracy and precision among representative sludge-thickness detection methods is presented in Table 4. This comparison highlights the improvements achieved by the present approach in terms of measurement deviation, repeatability, and stability under turbid conditions. As shown, while manual and optical techniques exhibit larger error margins and reduced consistency, and conventional ultrasonic methods often degrade in multiphase environments, the proposed system maintains superior accuracy (< 2% deviation) and precision (± 0.02 m), confirming its robustness for real-time sludge-interface monitoring in wastewater treatment operations.

**Table 4:** Comparison of measurement accuracy and precision among sludge-thickness detection methods.

Method	Typical Accuracy / Deviation	Precision / Repeatability	Major Error Sources	Performance in Turbid Media
**Manual rod insertion**	±3–5% (operator-dependent).	±0.03–0.05 m per reading.	Human error, limited resolution, inconsistent insertion depth.	Poor — affected by visual estimation and sludge disturbance.
**Optical / infrared sensors**	±2–3% in clear media; > 5% in turbid conditions.	±0.02 m (clean water).	Light scattering, fouling, color variation.	Low — strong signal attenuation and lens contamination.
**Conventional ultrasonic (time-of-flight)**	±2–3% under stable conditions; up to ±5% in high-turbidity tanks.	±0.02–0.04 m.	Multipath reflections, weak secondary echoes, temperature drift.	Moderate — accuracy degrades in heterogeneous sludge layers.
**Proposed energy-peak ultrasonic method**	**<**2 % mean deviation (R²> 0.98) compared with manual reference.	±0.02 m (1500 samples / scan).	Minimal — effectively suppresses pseudo-peaks and transient noise.	Excellent — stable performance across variable sludge concentration and flow conditions.

#### 5. 4.4. Limitations and Future Prospects.

A thorough analysis of the method’s performance constraints is essential to establish its credibility. Despite the promising results, several limitations and sources of error warrant consideration. The accuracy of the measurement is inherently tied to the speed of sound, which in our current system is compensated using a temperature-dependent empirical model. This model may not fully capture variations caused by complex dynamic factors such as salinity and suspended solids composition, introducing a potential source of error. Addressing this will require the future integration of targeted experimental calibration to enhance overall robustness.

Furthermore, signal integrity can be affected by probe fouling in the harsh sewage environment and by extreme hydrodynamic turbulence, which may generate transient pseudo-peaks. To ensure the credibility and reliability of the data, these challenges are mitigated through the multi-element array design, which provides spatial averaging against local anomalies, and the adaptive 3σ fault-threshold algorithm, which filters out physiologically implausible data spikes. Building upon this foundation, future work will focus on integrating direct in-situ sound velocity measurement to move beyond empirical models, thereby enhancing robustness against varying medium properties. The incorporation of machine learning algorithms is also planned to further refine peak identification in complex acoustic scenarios. Ultimately, extending this system into a multi-sensor fusion framework with edge-computing capabilities presents a strategic pathway toward developing more intelligent, adaptive, and sustainable urban sewage treatment systems.

#### 5. 4.5. Economic and operational analysis.

To further establish the industrial significance of the proposed system, it is essential to consider the trade-offs between its enhanced performance and operational factors such as cost, complexity, and maintenance.

(1) Cost-Benefit Trade-off: While the hardware cost of the proposed system is higher than that of a single-probe manual or conventional ultrasonic device due to the use of a three-sensor array and an ARM Cortex-M4 processor, these initial expenditures are offset by significant long-term operational savings. By enabling continuous, automated monitoring, the system eliminates the labor costs associated with manual rod insertion. Furthermore, as a retrofit solution that integrates with existing bridge scrapers and SCADA systems, the implementation cost is minimized relative to specialized standalone monitoring platforms.
(2) Complexity and Computational Load: The integration of median averaging filtering, interval maximum peak extraction, and 3σ adaptive correction increases the algorithmic complexity and requires higher processing power than simple time-of-flight methods. However, the chosen microprocessor platform handles these tasks in real-time with minimal latency. This increased “intelligence” at the edge significantly reduces the need for frequent manual recalibration, which is a major bottleneck for conventional systems in variable wastewater environments.
(3) Maintenance Requirements: Compared to optical or infrared sensors, which suffer from rapid lens fouling and require frequent manual cleaning in opaque sludge, the submerged ultrasonic transducers are inherently more resistant to environmental contamination. The system’s robustness against suspended solids and microbubbles further extends the maintenance intervals, promising a lower total cost of ownership (TCO) in municipal wastewater treatment applications.

## 6. Conclusions and future work

To address the limitations of existing sludge interface meters, specifically their limited interference resistance and significant variations in measurement accuracy within wastewater settling tank environments, a comprehensive technical solution has been developed. The principal research outcomes are summarized as follows:

(1) An adaptive sludge thickness detection system was engineered with an operational range of 3–10 meters, utilizing an ultrasonic pulse interval of 2.5 μs. Experimental validation at a 4-meter scale with 1,500 measurement points demonstrated stable bottom-peak signal detection. The system accurately quantified sludge thickness within 0.5–3 meters, and when validated against the manual rod insertion method, it demonstrated high accuracy with measurement deviations consistently below 2%, and a precision variance of ±0.02 m. Furthermore, comparative evaluations confirmed its superiority over conventional optical and time-of-flight methods in suppressing noise within highly turbid multi-phase environments.
(2) Implementation of a median averaging filter effectively eliminated transient noise in complex liquid matrices. Sampling cohorts of 50 data points achieved optimal noise reduction, aligning with signal processing methodologies documented in ultrasonic measurement systems.

The theoretical robustness of this filter against impulsive, non-Gaussian disturbances and the parameter-selection rationale for the 50-point cohort have been clarified in the revised Methodology and Results sections.

(3) The proposed algorithm successfully extracted secondary peaks obscured by acoustic interference. Empirical optimization established a site-specific interval width of 250 position points (corresponding to a physical depth of ~0.915 m) for peak resolution. While this parameter enhanced measurement reliability under the turbulent conditions of the tested facility, it reflects the specific physical thickness of the local transition zone and must be recalibrated for applications in disparate tank configurations or hardware sampling rates.
(4) An adaptive fault threshold algorithm was applied to rectify continuous output anomalies. This method repaired erroneous detection data and achieved robust fitting performance, corroborating the importance of real-time data correction mechanisms in sludge interface monitoring.

For practical implementation, the proposed system is designed for seamless integration into existing wastewater treatment infrastructure. It can be retrofitted onto bridge scrapers and its data integrated into central SCADA systems, promising benefits such as enhanced operational efficiency and cost savings. However, challenges including site-specific calibration and probe maintenance in harsh environments need to be addressed for widespread adoption.

Future research will focus on several directions to further enhance the system: integrating machine learning algorithms for robust peak identification, employing multi-sensor data fusion for improved reliability, developing real-time in-situ sound velocity calibration to minimize errors, and exploring edge-computing for distributed intelligence. Furthermore, to move beyond current empirical temperature compensations, future work will focus on developing a coupled multi-parameter acoustic model. This theoretical framework will explicitly link the speed of sound and acoustic attenuation coefficients (scattering and absorption) to vertical temperature gradients and local sludge concentrations. By establishing a deterministic relationship between the multiphase medium properties and the echo energy profile, the model aims to provide more rigorous compensation for signal attenuation and velocity variations under complex thermodynamic and hydrodynamic conditions. These advancements are expected to contribute significantly to the development of more adaptive, efficient, and sustainable urban sewage treatment systems.

It should be noted that the present study validated the proposed method using data from a single sedimentation tank at one wastewater treatment plant. Future work should include testing across multiple tanks and diverse plant environments to further verify and enhance the generalizability of the findings.

## Supporting information

S1 File Data from the Mafang Wastewater Treatment Plant, January 4, 2022. (XLSX)

S2 File Data from the Mafang Wastewater Treatment Plant, January 5, 2022. (XLSX)

S3 File Data from the Mafang Wastewater Treatment Plant, January 6, 2022. (XLSX)
